# Optimization of Infiltration and Drying Processes for Pre-Gelatinized Hulless Barley Rice: Effects on Quality, Texture, and Bioactive Compound Retention

**DOI:** 10.3390/foods15193480

**Published:** 2026-09-29

**Authors:** Wenwen Lv, Yi Zhang, Maoling Tan, Yanan Cao, Yuanhang Ren, Jian Li, Lianxin Peng

**Affiliations:** 1 Key Laboratory of Coarse Cereal Processing, Ministry of Agriculture and Rural Affairs, Sichuan Province Engineering Technology Research Center of Coarse Cereal Industrialization, School of Food and Biological Engineering, Chengdu University, Chengdu 610106, China; lvwenwen@cdu.edu.cn (W.L.);; 2 Ganzi Academy of Agricultural Sciences, Kangding 626000, China

**Keywords:** hulless barley, pre-gelatinization, infiltration, drying optimization, bioactive compounds

## Abstract

Hulless barley (*Hordeum vulgare* L. *var. nudum*) is a nutritious cereal but suffers from prolonged cooking and asynchronous gelatinization with rice. Pre-gelatinization technology can address these issues, yet optimal processing parameters are needed to preserve its bioactive components. This study optimized the infiltration and drying processes for pre-gelatinized hulless barley rice. Infiltration cycles and durations were evaluated using sensory scores, texture analysis, and gelatinization degree; drying temperatures (80–160 °C) and times were optimized based on quality and nutrient retention. Secondary infiltration for 4 h significantly outperformed traditional soaking, yielding higher retention of polyphenols (130.73 mg/100 g), flavonoids (50.01 mg/100 g), and β-glucan (2.96%). Drying at 80–100 °C for 90–120 min produced the best sensory and textural properties, maximal retention of polyphenols (128.46 mg/100 g), flavonoids (45 mg/100 g), β-glucan (3.1%), and reducing sugars (482.84 mg/100 g at 80 °C/120 min), and the highest DPPH and ABTS^+^ radical scavenging activities. Higher temperatures (≥120 °C) caused significant degradation of these functional components and reduced antioxidant capacity. The optimal combination—secondary infiltration for 4 h followed by drying at 80–100 °C for 90–120 min—effectively balances processing efficiency with nutritional and functional quality. This optimized process provides a practical technical solution for large-scale production of high-quality pre-gelatinized hulless barley rice.

## 1. Introduction

Hulless barley (*Hordeum vulgare* L. *var. nudum Hook. f.*), also known as naked barley, is a distinctive cereal crop of the genus Hordeum (family Poaceae) and ranks among the highest-altitude crops cultivated worldwide. It is predominantly distributed across the Qinghai–Tibetan Plateau in China, as well as the western regions of Sichuan, Yunnan, and Gansu provinces, and is also cultivated in neighboring Himalayan countries, including Bhutan and Nepal [1]. Owing to its naked caryopsis and ease of dehulling, hulless barley offers greater convenience in grain processing and direct consumption compared with common barley. It has served as the traditional staple food for generations of inhabitants of the Qinghai–Tibetan Plateau and is revered by the Tibetan people as the “king of highland grains” [2]. Characterized by exceptional tolerance to cold, drought, and low soil fertility, hulless barley has become an indispensable food crop in high-altitude regions, playing an irreplaceable role in ensuring regional food security.

As a premium cereal crop, hulless barley possesses a nutritional profile consistent with a healthy dietary pattern characterized by high protein, high fiber, high vitamins, low fat, and low sugar [3,4]. Its protein and lysine contents exceed those of most other cereal crops, playing an important role in promoting growth and development and enhancing immune function [5,6,7]. Additionally, hulless barley is rich in dietary fiber, particularly β-glucan—often referred to as a “health guardian”—which has been extensively demonstrated to exert multiple physiological functions, including lowering blood glucose and blood lipids, enhancing immunity, and improving gut health [8,9,10,11]. Furthermore, hulless barley contains abundant polyphenolic compounds (e.g., ferulic acid, proanthocyanidins), γ-aminobutyric acid (GABA), and minerals such as calcium, iron, zinc, and magnesium. These functional components exhibit significant positive effects in antioxidant, anti-inflammatory, and neuroregulatory activities, as well as in maintaining cardiovascular health and bone metabolism [12,13,14,15]. Owing to this comprehensive and balanced nutritional composition, hulless barley not only satisfies basic energy requirements but also demonstrates considerable potential in promoting health among modern populations.

Hulless barley can be processed into a wide variety of food products, ranging from traditional preparations such as tsampa and hulless barley wine to innovative developments driven by the modern food industry. However, like other whole grains, hulless barley exhibits a hard pericarp and a dense starch granule structure, which result in a prolonged cooking time and a technical bottleneck wherein it fails to gelatinize synchronously when cooked together with rice [16]. To address this issue, grain ripening (or pre-gelatinization) technology has been developed. This process involves pretreatment methods such as steaming, microwave irradiation, or conditioning to induce partial starch gelatinization, thereby significantly reducing cooking time, improving palatability, and enhancing the digestibility of nutrients [17,18,19]. Based on this technology, pre-gelatinized hulless barley rice has been developed, which achieves simultaneous cooking with rice. This product combines convenience with high nutritional value, meeting the urgent consumer demand for healthy and convenient foods in a fast-paced lifestyle, and thus holds considerable market potential [20,21].

Progress has been achieved in the processing technology of pre-gelatinized rice, such as the simple operation but low efficiency of the steaming method and the high efficiency yet high equipment cost and susceptibility to uneven heating associated with microwave pre-gelatinization technology [22,23]. Against this backdrop, optimizing the processing parameters to balance product quality, nutrient retention, and production efficiency has become a key issue for industrial development. Particularly for hulless barley, which is rich in heat-sensitive and water-soluble functional components, how to maximize the retention of bioactive substances such as polyphenols, flavonoids, and β-glucan during the pre-gelatinization process represents a core scientific question for enhancing the added value of the product.

Therefore, this study focuses on two key process steps in the production of pre-gelatinized hulless barley rice: soaking and drying. To address the challenges of water-soluble nutrient loss during conventional soaking and the unclear effects of drying conditions on product texture and functional properties, this paper aims to systematically investigate the influence of soaking conditions (e.g., the balance between water absorption and nutrient retention), as well as different drying temperatures and durations, on the sensory quality, textural properties, degree of gelatinization, and major functional component contents of pre-gelatinized hulless barley rice. Through comprehensive analysis of the above evaluation indicators, we intend to identify the optimal combination of processing parameters. This will provide a scientific basis and technical support for developing a novel pre-gelatinized hulless barley rice product that combines excellent eating quality with high nutritional value, thereby enriching the variety of hulless barley products, enhancing their market competitiveness, and promoting the sustainable development of the hulless barley industry.

## 2. Materials and Methods

### 2.1. Materials

Kangding black hulless barley (*Hordeum vulgare* L. *var. nudum Hook. f.*) was provided by the Key Laboratory of Coarse Cereal Processing, Ministry of Agriculture and Rural Affairs, Chengdu University. Potassium hydroxide, methanol, hydrochloric acid, iodine, and potassium iodide were all of analytical grade and purchased from Chengdu Kelong Chemical Co., Ltd. (Chengdu, China).

### 2.2. Investigation of the Infiltration Process for Hulless Barley

To develop a precise moisture control method for hulless barley seeds, this study employed an infiltration technique. Under room temperature conditions, uniformly sized and disease-free hulless barley seeds were placed in distilled water. Samples were taken at 0, 1, 2, 3, 4, 5, 6, 7, and 8 h, respectively. After removing surface moisture with absorbent paper, the seeds were weighed to calculate the water absorption rate, and a dynamic seed water absorption curve was plotted. Infiltration, as a technique that regulates the degree of contact between seeds and water, provides the appropriate amount of water required for germination and avoids hypoxia and nutrient loss caused by excess water, thereby activating enzyme activity and promoting uniform and healthy germination. Unlike complete immersion (soaking), infiltration places greater emphasis on controlling the water uptake process. The time–water absorption rate curve obtained in this study can provide a reference for the appropriate amount of water to be added at different time points in subsequent infiltration processes, ensuring that seed water absorption precisely meets germination requirements.

$$Wt=\frac{Mt-M_{0}}{M_{0}}\times 100\%$$

M*t*: mass of hulless barley after water absorption (with surface moisture blotted dry), M_0_: initial mass of hulless barley, W*t*: water absorption rate of hulless barley.

### 2.3. Process Flow for Pre-Gelatinized Hulless Barley Rice

Grains of hulless barley with uniform size and no disease symptoms were manually screened. The grains were subjected to precise primary–secondary infiltration according to the pre-established water absorption curve, or conventional full-immersion soaking for the target duration to achieve the desired water uptake. Upon completion of infiltration or soaking, the grains were rapidly rinsed with deionized water for 30 s to remove soluble residues on the kernel surface. Afterwards, the grains were spread in a uniform single layer on stainless steel trays for hot air drying, with the drying temperature and duration strictly set in accordance with the experimental design. After drying, samples were taken out and force-air cooled to room temperature (25 °C) in a clean and dust-free environment. Once fully cooled, the samples were immediately vacuum packaged using aluminum plastic composite bags to isolate them from oxygen and ambient moisture and avoid oxidative deterioration of bioactive components. Stable pre-gelatinized hulless barley rice samples were thereby obtained for subsequent quality analysis. All processing treatments were performed in triplicate.

### 2.4. Optimization of the Infiltration Process for Pre-Gelatinized Hulless Barley Rice

#### 2.4.1. Optimization of Primary and Secondary Infiltration Times

First, the primary and secondary infiltration times were optimized. Primary infiltration refers to the first-stage moisture-regulating infiltration treatment of hulless barley grains; after completing primary infiltration, surface free water was removed with absorbent paper, and then the grains immediately underwent the second-stage infiltration treatment, which is defined as secondary infiltration. Compared with single-stage infiltration, the segmented primary–secondary infiltration mode can realize more uniform water penetration inside kernels, avoid excessive free water immersion occurring in traditional full soaking, reduce leaching loss of water-soluble bioactive components, and promote uniform starch gelatinization.

Both primary and secondary infiltration times were set at 1, 2, 3, and 4 h, resulting in the following treatment groups: 1–1, 1–2, 1–3, 1–4, 2–1, 2–2, 2–3, and 2–4. The quality of the cooked pre-gelatinized hulless barley rice was comprehensively evaluated using sensory evaluation, texture profile analysis, and degree of gelatinization measurement.

#### 2.4.2. Comparison of Infiltration and Soaking Processes

The optimized infiltration process was compared with the traditional soaking process using raw hulless barley grains. Differences between the infiltration and soaking processes were comprehensively investigated through sensory evaluation, degree of gelatinization, textural properties, and changes in water-soluble active components.

#### 2.4.3. Optimization of the Drying Process After Infiltration

Based on the optimal infiltration conditions, the temperature and time parameters of the drying process after infiltration were further optimized. The experimental design was as follows:

Drying temperatures: 80 °C, 100 °C, 120 °C, 140 °C, and 160 °C. Drying times: For each temperature, three time points were set based on actual conditions to ensure that the moisture content of the pre-gelatinized hulless barley fell within the specified range after processing:

80 °C: 90 min, 120 min, 150 min; designated as groups 80–90, 80–120, 80–150.

100 °C: 60 min, 90 min, 120 min; designated as groups 100–60, 100–90, 100–120.

120 °C: 50 min, 80 min, 110 min; designated as groups 120–50, 120–80, 120–110.

140 °C: 40 min, 60 min, 80 min; designated as groups 140–40, 140–60, 140–80.

160 °C: 30 min, 50 min, 70 min; designated as groups 160–30, 160–50, 160–70.

Sensory evaluation, degree of gelatinization, and texture profile analysis were used to select the optimal drying time at each temperature.

The optimal time combinations at each drying temperature were further compared using a multi-index evaluation approach, including functional component analysis (polyphenol and β-glucan contents), flavor component analysis (electronic nose and electronic tongue), and antioxidant capacity determination (DPPH and ABTS^+^ radical scavenging activities), to investigate the effects of drying temperature and time on the nutritional quality of the pre-gelatinized rice.

### 2.5. Sensory Evaluation of Pre-Gelatinized Hulless Barley Rice

The sensory scoring criteria for pre-gelatinized hulless barley rice were established with reference to the Chinese national standard GB/T 15682-2008 [24], with slight modifications. Ten panelists with a background in food science were recruited to form the scoring team. According to Table 1, the cooked hulless barley rice was evaluated for color, aroma after co-cooking with rice, taste, chewiness, and viscoelasticity. The total score was 100 points.

### 2.6. Determination of Nutritional Component Contents

The contents of moisture, fat, starch, and protein were determined according to the methods specified in the corresponding Chinese national standards.

Moisture content: Determined according to GB 5009.3-2016 [25] National Food Safety Standard—Determination of moisture in foods (direct drying method).

Fat content: Determined according to GB 5009.6-2016 [26] National Food Safety Standard—Determination of fat in foods (Soxhlet extraction method).

Protein content: Determined according to GB 5009.5-2016 [27] National Food Safety Standard—Determination of protein in foods (Kjeldahl method).

Starch content: Determined according to GB 5009.9-2023 [28] National Food Safety Standard—Determination of starch in foods.

### 2.7. Texture Analysis of Pre-Gelatinized Hulless Barley Rice

After co-cooking the pre-gelatinized hulless barley with rice, three grains of moderate size and uniform texture were selected and placed on the platform of a texture analyzer (TA-XT plus, Stable Micro Systems Ltd., Godalming, UK). Measurements were performed using the texture profile analysis (TPA) mode with a P36R probe. The test parameters were set as follows: trigger force 5 g; two cycles of compression; compression ratio 75%; pre-test and post-test speed 5 mm/min; compression speed 1 mm/s; and interval time between cycles 5 s.

### 2.8. Determination of Gelatinization Degree of Pre-Gelatinized Hulless Barley Rice

The gelatinization degree of pre-gelatinized hulless barley rice was determined based on the method of Liu et al. [29] with slight modifications. A 0.2 g sample of dried and ground pre-gelatinized hulless barley powder was weighed and mixed with 98 mL of distilled water, followed by the addition of 2 mL of 10 mol/L KOH solution, with gentle stirring for 5 min. The resulting suspension was centrifuged. Then, 1 mL of the supernatant was collected, mixed with 0.4 mL of 0.5 mol/L HCl solution, and diluted with distilled water to a final volume of 10 mL. Subsequently, 0.1 mL of iodine reagent (prepared by dissolving 1 g of iodine and 4 g of potassium iodide in 100 mL of water) was added. After thorough mixing, the absorbance was measured at 600 nm using a spectrophotometer (UV-2600 UV-Vis spectrophotometer, Shimadzu Corporation, Kyoto, Japan) and recorded as A_1_. Separately, another 0.2 g sample powder was treated with 95 mL of distilled water and 5 mL of 10 mol/L KOH solution, neutralized with 1.0 mL of 0.5 mol/L HCl solution, and thoroughly mixed. The absorbance was measured at 600 nm and recorded as A_2_. The gelatinization degree for each sample was calculated from the ratio of the absorbances obtained under the two conditions.

$$\mathrm{Gelatinization}\;\mathrm{degree}=\frac{A_{1}}{A_{2}}\times 100\%$$

### 2.9. Determination of Total Phenolic Content of Pre-Gelatinized Hulless Barley Rice

The total phenolic content of pre-gelatinized hulless barley rice was determined using a Plant Total Phenol Assay Kit (96-well plate, purchased from Beijing Solarbio Science & Technology Co., Ltd., Beijing, China). The pre-gelatinized hulless barley rice was ground into powder and passed through a 60-mesh sieve. The total phenolic content was then measured according to the manufacturer’s instructions. Each sample was analyzed in triplicate.

### 2.10. Determination of β-Glucan Content of Pre-Gelatinized Hulless Barley Rice

The β-glucan content was quantified with a Mixed-Linkage β-Glucan Assay Kit (96-well plate format, Megazyme International, Bray, Ireland) based on the official enzymatic assay procedure provided by the manufacturer. Briefly, pre-gelatinized hulless barley rice samples were ground into fine powder and sieved through a 60-mesh screen. Then, a 100 mg sample powder was accurately weighed and transferred into a test tube, followed by the addition of 2.0 mL of sodium phosphate buffer (20 mM, pH 6.5). The mixture was vortexed and incubated in a water bath at 100 °C for 5 min to promote starch gelatinization. After cooling the tube to 50 °C, 0.2 mL of lichenase solution was added, vortexed, and incubated at 50 °C for 60 min with intermittent shaking to completely hydrolyze the mixed-linkage β-glucan. Subsequently, 2.5 mL of sodium acetate buffer (200 mM, pH 4.0) was added to terminate the enzymatic reaction. The tube was vortexed and centrifuged at 3000× *g* for 10 min at room temperature. An aliquot of 0.1 mL of the resulting supernatant was transferred to a new test tube, and 0.1 mL of β-glucosidase working solution was added. After thorough mixing, the mixture was incubated at 50 °C for 10 min. For the blank control, 0.1 mL of supernatant was mixed with 0.1 mL of sodium acetate buffer (200 mM, pH 4.0) without β-glucosidase. Next, 1.5 mL of GOPOD reagent was added to both sample and blank tubes, mixed well, and incubated at 50 °C for 20 min. The absorbance of each solution was measured at 510 nm using a microplate reader. The β-glucan content was calculated according to the calculation formula supplied in the kit manual. Each sample was analyzed in triplicate.

### 2.11. Determination of Total Flavonoid Content of Pre-Gelatinized Hulless Barley Rice

The total flavonoid content was quantified via the aluminum salt colorimetric method using rutin as the reference standard to prepare standard working solutions. The linear regression equation of the standard curve was established as y = 0.828x + 0.041, with a coefficient of determination R^2^ = 0.9989. Precisely 1 mL of the cooked hulless barley rice sample extract was pipetted and diluted 10-fold, followed by absorbance measurement at 510 nm. The total flavonoid content of the sample was calculated based on the standard curve, and the results were expressed in mg/100 g. All samples were analyzed in triplicate.

### 2.12. Determination of Reducing Sugar Content of Pre-Gelatinized Hulless Barley Rice

A 0.1 g sample of dried pre-gelatinized hulless barley powder was weighed and mixed with 5 mL of distilled water. The mixture was shaken and placed in a 50 °C water bath for 30 min, then centrifuged at 8000 r/min for 20 min. The supernatant was collected for analysis. The reducing sugar content was determined using the 3, 5-dinitrosalicylic acid (DNS) method: 0.1 mL of the supernatant was transferred into a centrifuge tube, followed by the addition of 0.4 mL of distilled water and 1.5 mL of DNS chromogenic reagent. After thorough mixing, the tube was heated in a boiling water bath for 5 min, then cooled, and the absorbance was measured at 540 nm. The reducing sugar content was calculated based on a standard curve (y = 1.3776x + 0.0363, R^2^ = 0.999).

### 2.13. Intelligent Sensory Analysis of Pre-Gelatinized Hulless Barley Rice

#### 2.13.1. Electronic Tongue Analysis

The taste profile of pre-gelatinized hulless barley rice was determined using an electronic tongue (ASTREE V 5.1, Alpha M.O.S., Toulouse, France) following the method described by Liu et al. [30] with slight modifications. The sensors of the electronic tongue were used to measure various taste attributes, including sourness, sweetness, bitterness, saltiness, and umami. The measurement program was set as follows: electrode cleaning for 90 s, buffer cleaning for 120 s, buffer cleaning for 120 s, stabilization for 30 s, sample testing for 30 s, buffer cleaning for 3 s, buffer cleaning for 3 s, and aftertaste determination for 30 s. Each sample was measured in five replicate cycles. The sensor performance characteristics are presented in Table 2.

#### 2.13.2. Electronic Nose Analysis

The electronic nose (PEN3, Arisense, Schwerin, Germany) was used to analyze the flavor profile of pre-gelatinized hulless barley rice, following the method described by Jiang et al. [31] with slight modifications. The measurement parameters were set as follows: cleaning time 90 s, detection time 150 s, and carrier gas flow rate 500 mL/min. Each sample was measured in five replicates. The volatile compounds were detected using different sensors, and the sensor performance characteristics are presented in Table 3.

### 2.14. Determination of Antioxidant Activity

The hulless barley rice powder was passed through a 60-mesh sieve. Briefly, 0.5 g of the sieved sample powder was accurately weighed into a 50 mL centrifuge tube, and 30 mL of 70% methanol solution was added. Ultrasonic extraction was performed at room temperature with a frequency of 100 Hz for 30 min. The mixture was centrifuged at 4000 r/min for 10 min, and the supernatant extract was collected. The residual pellet was re-extracted twice under identical conditions. The supernatants from the three extractions were combined and diluted to a final volume of 100 mL with 70% methanol solution to prepare a sample working solution at 5 mg/mL.

The supernatant was serially diluted with 80% methanol solution to prepare sample working solutions at concentrations of 0.02, 0.04, 0.06, 0.08, 0.1, 0.2, 0.4, 0.6, 0.8 and 1.0 mg/mL. The VC solution was used as the positive control, and the half-maximal inhibitory concentration (IC_50_) was calculated. The DPPH radical scavenging assay was performed with slight modifications according to the method described by Uzkuç et al. [32]. Briefly, 2 mL of the sample working solution was accurately pipetted and mixed with 2 mL of DPPH solution, followed by thorough shaking. The mixture was incubated at room temperature in the dark for 30 min, and the absorbance was measured at 517 nm and recorded as Ai. An equal volume of 80% methanol solution was used to replace the DPPH solution, and the absorbance was determined using the same procedure and denoted as Aj. An equal volume of 80% methanol solution was used to replace the sample working solution, and the absorbance was measured identically and recorded as Ac. The VC solution was set as the positive control and subjected to the same measurement procedure for DPPH radical scavenging activity. All assays were performed in triplicate. The DPPH radical scavenging rate was calculated using the following formula:

$$\mathrm{DPPH}\;\mathrm{radical}\;\mathrm{scavenging}\;\mathrm{rate}\;\left(\%\right)=(1-\frac{\mathrm{Ai}-\mathrm{Aj}}{\mathrm{Ac}})\times 100$$

The ABTS radical scavenging assay was carried out with minor modifications based on the method reported by Segoviano-Leon et al. [33]. Equal volumes of 7 mmol/L ABTS solution and 2.5 mmol/L potassium persulfate solution were mixed and kept in the dark at room temperature for 24 h to prepare the ABTS stock solution. Before use, an appropriate amount of the ABTS stock solution was diluted with 95% ethanol until its absorbance reached 0.70 ± 0.02 at 734 nm to obtain the ABTS working solution. Briefly, 0.1 mL of the serially diluted sample solution was added to a 5 mL centrifuge tube, mixed thoroughly with 3.9 mL of ABTS working solution, and incubated in the dark at room temperature for 6 min. The absorbance was measured at 734 nm and recorded as Ai. An equal volume of absolute ethanol was used to replace the ABTS working solution, and the absorbance was detected following the same procedure and marked as Aj. An equal volume of 80% methanol solution was used to replace the sample solution, and the absorbance was measured identically and denoted as Ac. A VC solution was used as the positive control, and the ABTS radical scavenging activity was determined by the same procedure. All assays were performed in triplicate. The ABTS radical scavenging rate was calculated according to the following formula:

$$\mathrm{ABTS}\;\mathrm{radical}\;\mathrm{scavenging}\;\mathrm{rate}\;\left(\%\right)=(1-\frac{\mathrm{Ai}-\mathrm{Aj}}{\mathrm{Ac}})\times 100$$

### 2.15. Statistical Analysis

All experimental data are expressed as mean ± standard deviation. Two-way analysis of variance (ANOVA) was conducted for group comparisons. Significant differences among treatments were determined by Tukey’s HSD post-hoc multiple-comparison test, with *p* < 0.05 regarded as statistically significant. Tukey’s HSD test inherently performs adjustment for multiple comparisons. All statistical calculations were completed using SPSS 26.0 software. Different lowercase superscript letters in tables and figures indicate significant differences.

## 3. Results and Discussion

### 3.1. Changes in Water Absorption Rate of Hulless Barley During Infiltration

**Figure 1:**
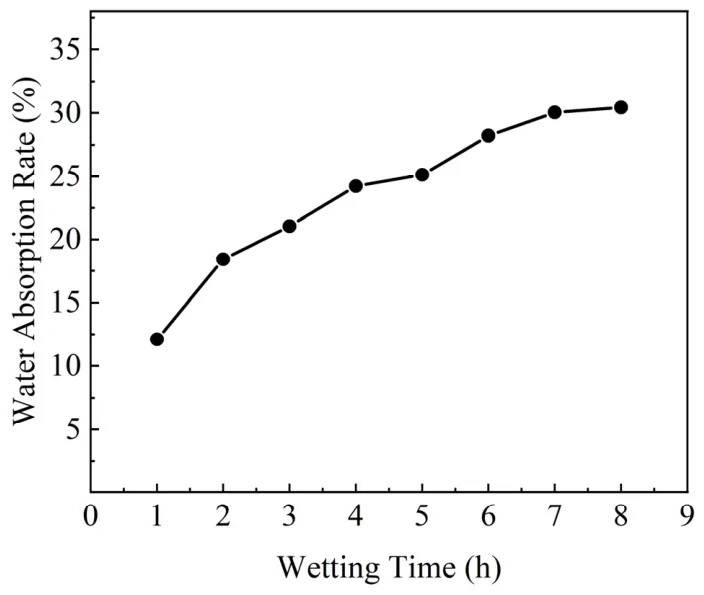
Water absorption rate curve of hulless barley.

Figure 1 shows the changes in the water absorption rate of hulless barley as a function of infiltration time. The results indicated that the water absorption rate gradually increased with prolonged infiltration time, exhibiting a trend of rapid increase followed by a slower phase. At the initial stage of infiltration, the water absorption rate was high, reflecting a strong water uptake capacity in hulless barley. Between 2 and 5 h, the absorption rate gradually decreased. After 5 h, the water absorption rate leveled off. This curve reveals the dynamic process of water absorption in hulless barley, providing data support for the infiltration treatment and processing of hulless barley. During the infiltration process, water absorption was relatively rapid within the first 1–3 h, and the absorption rate became gentle after 4 h. Moreover, excessively long infiltration times are not suitable for practical production and cannot meet application requirements. Therefore, 4 h was selected as the maximum infiltration time for subsequent experiments.

### 3.2. Optimization of Primary and Secondary Infiltration Times

#### 3.2.1. Gelatinization Degree and Sensory Scores

**Figure 2:**
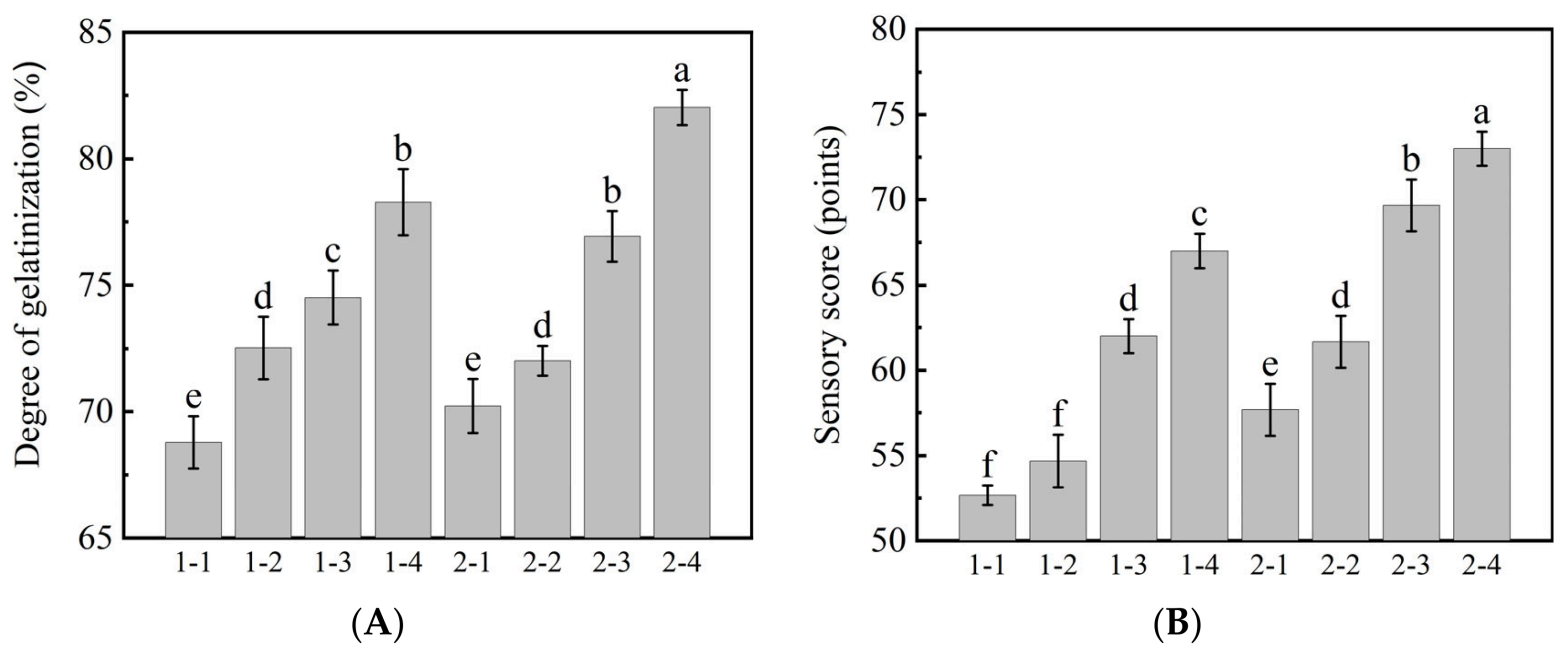
Gelatinization degree (**A**) and sensory scores (**B**) of hulless barley under different primary and secondary infiltration times. Different superscript letters indicate significant differences among groups (*p* < 0.05).

Figure 2A presents the changes in the gelatinization degree of hulless barley under different combinations of primary and secondary infiltration times. As shown in the figure, the gelatinization degree gradually increased with prolonged infiltration time, exhibiting a clear upward trend. At shorter primary and secondary infiltration times, the gelatinization degree was relatively low, indicating limited gelatinization under brief infiltration. However, as both primary and secondary infiltration times were extended, the gelatinization degree progressively increased, reaching a maximum of 82.03% under the longer time conditions. These results suggest that appropriate extension of infiltration time can significantly enhance the gelatinization degree of hulless barley, thereby improving its processing characteristics and final product quality. Therefore, rational optimization of the infiltration time combination is conducive to enhancing the adaptability of hulless barley in food processing, leading to better performance in subsequent production steps.

Figure 2B illustrates the sensory scores of hulless barley rice under different combinations of primary and secondary infiltration times. The results showed that the sensory scores generally increased with optimized infiltration times, and the different time combinations had a significant effect on the scores. At shorter primary and secondary infiltration times, the scores were lower; however, with appropriate prolongation of infiltration time, the scores gradually improved, eventually reaching the highest value at a secondary infiltration time of 4 h. This indicated that reasonable optimization of the infiltration time combination can significantly enhance the sensory quality of hulless barley, providing a scientific basis for optimizing the infiltration process in practical production.

#### 3.2.2. Textural Properties

As shown in Table 4, the hardness of hulless barley gradually decreased with prolonged infiltration time, reaching the lowest value of 9845.98 ± 120.97 g at a secondary infiltration time of 4 h, indicating that extended infiltration promotes water penetration and softens cell walls. At primary infiltration times of 1 and 2 h, insufficient water penetration resulted in relatively high hardness; at 3 h, water entered the grains but was unevenly distributed, leading to a transient increase in hardness due to the compact structure; after 4 h of primary infiltration, water was more uniformly distributed, and hardness slightly decreased. The secondary infiltration further equilibrated moisture, leading to more uniform softening of the barley structure and effectively reducing hardness.

The springiness of hulless barley showed relatively little overall variation, which was influenced by moisture distribution and protein structure. Under short infiltration times, insufficient protein hydration resulted in lower springiness; as water gradually penetrated, protein hydration increased, enhancing springiness. The secondary infiltration further balanced moisture, avoiding a decrease in springiness caused by localized over-hydration while maintaining good textural and processing properties.

The chewiness of hulless barley generally decreased, with values of 136.75 ± 15.40 g at 1 h of primary infiltration, decreasing to 121.75 ± 18.88 g at 3 h, and slightly rebounding to 128.76 ± 13.57 g at 4 h; the lowest value of 56.53 ± 7.20 g was observed at 4 h of secondary infiltration, representing a 58.6% reduction compared to the 1 h primary infiltration. This may be attributed to the fact that at short infiltration times, water mainly remained on the surface, resulting in greater chewing resistance. With extended time, water penetrated into the interior, causing starch swelling and texture softening, thereby reducing chewiness [34]. The secondary infiltration promoted uniform moisture distribution, making the grains softer, effectively lowering chewing resistance and significantly improving the mouthfeel and eating quality of the pre-gelatinized rice.

### 3.3. Effects of Infiltration and Soaking Followed by Drying on the Quality of Pre-Gelatinized Hulless Barley Rice

#### 3.3.1. Gelatinization Degree and Sensory Evaluation

**Figure 3:**
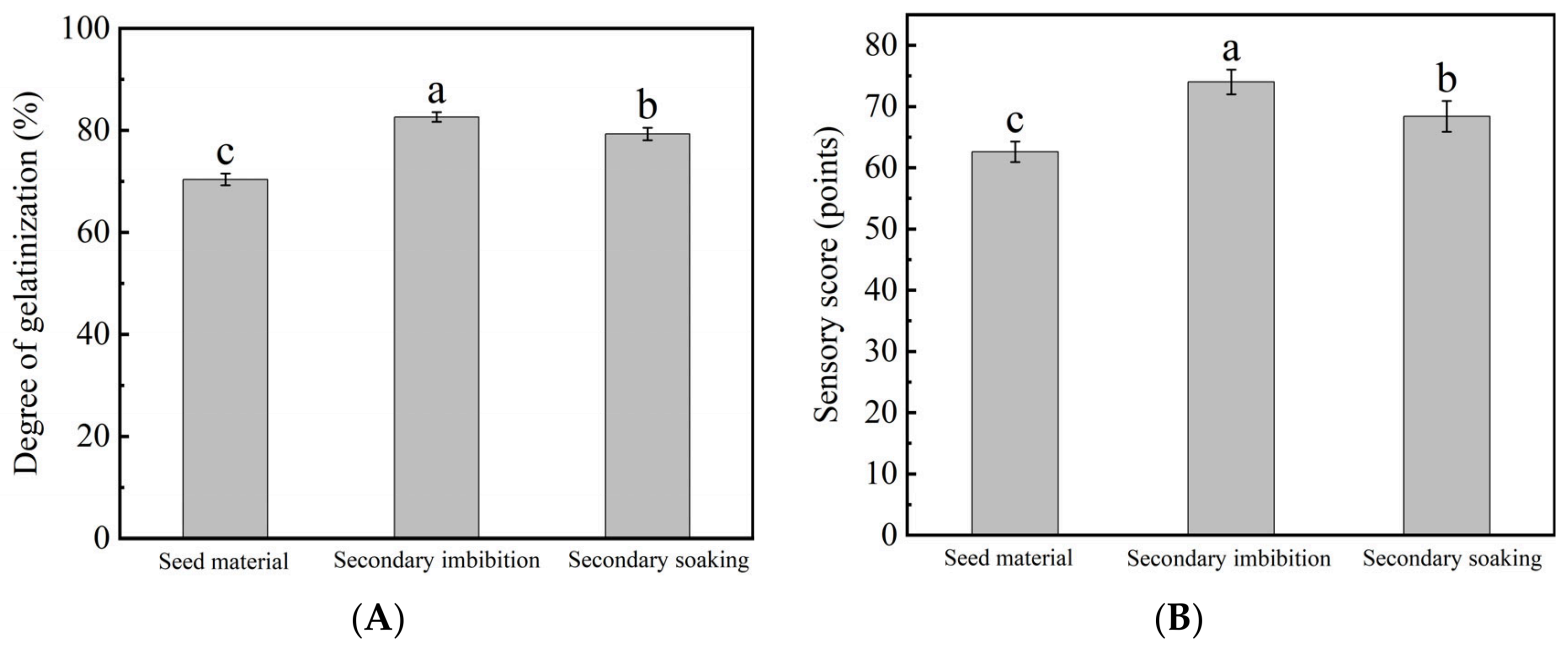
Gelatinization degree (**A**) and sensory scores (**B**) of pre-gelatinized hulless barley rice under different processing methods. Different superscript letters indicate significant differences among groups (*p* < 0.05).

Figure 3A shows that different processing methods had significant effects on the gelatinization degree of hulless barley. The raw hulless barley exhibited the lowest gelatinization degree (70.37%), likely because its internal structure remained largely ungelatinized without any pretreatment. Secondary soaking for 4 h increased the gelatinization degree to 79.27%, but this was still inferior to secondary infiltration for 4 h, presumably due to insufficient water absorption uniformity and efficiency. The highest gelatinization degree (82.61%) was observed in the secondary infiltration for 4 h treatment, which was significantly superior to the other methods. This indicates that secondary infiltration can more effectively enhance the gelatinization efficiency of hulless barley, providing a better raw material basis for subsequent processing.

Figure 3B illustrates the effects of different processing methods on the sensory scores of pre-gelatinized hulless barley rice. The results showed that the secondary infiltration for 4 h group achieved the highest sensory score (74 points), which was significantly higher than those of the secondary soaking for 4 h group (68.4 points) and the raw material group (62.6 points). The infiltration process improved the moisture uniformity of the grains, resulting in a better texture and mouthfeel after cooking. In contrast, the secondary soaking for 4 h group yielded rice with inferior texture and uniformity compared to the infiltration treatment. The raw material group had the lowest sensory score (62.6 points), indicating that unoptimized pre-gelatinized hulless barley rice had notable defects in taste and texture, possibly due to uneven moisture distribution leading to higher grain hardness. Overall, secondary infiltration for 4 h significantly improved the sensory quality of pre-gelatinized hulless barley rice.

#### 3.3.2. Determination of Basic Nutritional Components

Table 5 presents the effects of different processing treatments on the basic nutritional components of pre-gelatinized hulless barley rice. Compared with the raw hulless barley (moisture content 13.49%), the moisture contents after secondary infiltration and secondary soaking decreased to 8.65% and 8.68%, respectively, indicating that infiltration or soaking combined with drying can effectively reduce moisture content and improve product storage stability. The fat content showed no significant difference among the treatments, with values of 1.78% and 1.58% after secondary infiltration and secondary soaking, respectively, which were slightly lower than that of raw hulless barley (1.80%), possibly due to the loss of minor water-soluble lipids. The protein content decreased after all treatments, with values of 10.67% and 10.23% after secondary infiltration and secondary soaking, respectively, compared with 11.50% in the raw material, which may be attributed to the dissolution and loss of some water-soluble proteins during the infiltration or soaking process. The starch content decreased to 60.2% and 60.5% after secondary infiltration and secondary soaking, respectively, which was lower than that of the raw hulless barley (64.80%), suggesting that some oligosaccharides may have leached out during water penetration, leading to a reduction in starch content. Comprehensive analysis indicated that both secondary infiltration and secondary soaking led to decreases in the moisture, protein, and starch contents of pre-gelatinized hulless barley rice, whereas the fat content changed only slightly. The secondary infiltration process was superior to secondary soaking in terms of nutrient retention, demonstrating that infiltration can effectively reduce the loss of water-soluble nutrients and improve the nutritional quality of the pre-gelatinized rice.

#### 3.3.3. Textural Properties

Table 6 shows that different processing treatments had significant effects on the textural properties of pre-gelatinized hulless barley rice. The raw material exhibited the highest hardness (18,290.24 ± 420.52 g), indicating a firm texture that was not conducive to consumption. The secondary infiltration for 4 h treatment significantly reduced hardness, achieving the best softening effect. In contrast, the secondary soaking for 4 h treatment resulted in a hardness of 11,937.74 ± 305.24 g, which, although reduced, remained higher than that of the secondary infiltration group, suggesting that the infiltration process more effectively promoted uniform moisture distribution within the grains, thereby enhancing the softening effect [35]. Springiness varied only slightly among the treatments, ranging from 0.08 to 0.10 mm, indicating that both infiltration and soaking had a relatively limited impact on springiness; however, the secondary infiltration for 4 h treatment maintained appropriate springiness while reducing hardness, contributing to a more stable grain texture. The raw material had the highest chewiness (136.19 ± 45.4 g), implying that untreated hulless barley required greater masticatory force. The secondary infiltration for 4 h treatment significantly reduced chewiness to 56.53 ± 7.20 g, consistent with the decreasing trend in hardness. The chewiness of the secondary soaking for 4 h treatment was 137.68 ± 14.4 g, which, though lower than that of the raw material, was still considerably higher than that of the secondary infiltration group, indicating that the infiltration process had a greater advantage in reducing chewing resistance. Overall, the secondary infiltration for 4 h treatment not only reduced hardness and chewiness but also preserved adequate springiness, resulting in a more uniform and stable grain texture, thereby effectively improving the eating quality of pre-gelatinized hulless barley rice.

#### 3.3.4. Contents of Polyphenols, Flavonoids, and β-Glucan

**Figure 4:**
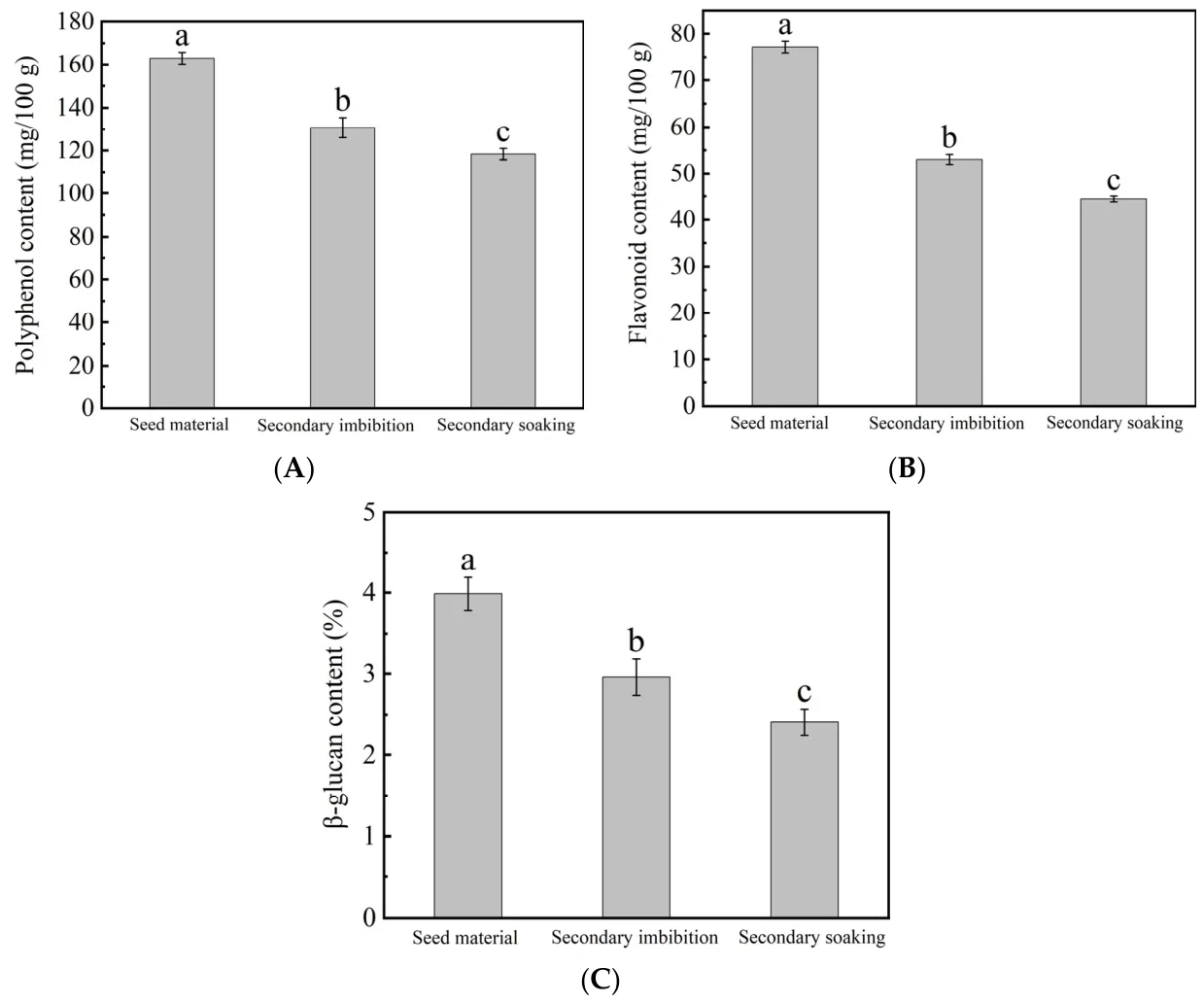
Polyphenol (**A**), flavonoid (**B**) and β-glucan (**C**) contents of cooked barley rice under different processes. Different superscript letters indicate significant differences among groups (*p* < 0.05).

Figure 4A shows that the raw material exhibited the highest polyphenol content (162.97 mg/100 g), indicating that unprocessed hulless barley retained the greatest amount of natural polyphenols. After secondary soaking for 4 h, the polyphenol content decreased to 118.35 mg/100 g. The soaking process allowed free water penetration, disrupting cell structures and releasing more polyphenols, while potentially activating polyphenol oxidase (PPO), which accelerates degradation [36,37]. In contrast, after secondary infiltration for 4 h, the polyphenol content was 130.73 mg/100 g. Although some water-soluble polyphenols dissolved into the infiltration medium, the infiltration process can better control water penetration and reduce polyphenol loss [38]. In summary, secondary infiltration better preserves the polyphenolic components of hulless barley, mainly due to more effective control of water-soluble component leaching and better maintenance of cell integrity.

Figure 4B indicates that the raw material had the highest flavonoid content (77.16 mg/100 g), suggesting that unprocessed hulless barley retained more natural flavonoids. Flavonoids possess strong antioxidant activity, but their content can be affected by water migration, dissolution loss, and oxidative degradation during processing [39]. After secondary infiltration for 4 h, the flavonoid content decreased to 50.01 mg/100 g, mainly due to the dissolution and loss of some flavonoids during the infiltration process. The infiltration process can precisely control the water absorption rate, limit flavonoid leaching, and effectively retain flavonoids. In comparison, after secondary soaking for 4 h, the flavonoid content decreased to 44.41 mg/100 g. The soaking process led to more significant flavonoid loss, consistent with previous reports linking free water exposure to greater losses of flavonoids in cereal grains. Furthermore, polyphenols may interact with starch molecules to form complexes, which helps reduce the loss of phenolic compounds during grain processing, as reported in starch-polyphenol complex systems [40].

Figure 4C reveals that the raw material had the highest β-glucan content (3.99%), indicating that unprocessed hulless barley can better retain its natural dietary fiber. During processing, β-glucan was reduced due to dissolution loss and temperature effects. After secondary infiltration for 4 h, the content decreased to 2.96%, indicating that some β-glucan entered the aqueous phase due to its water-soluble nature; however, the infiltration process can better control water penetration and reduce loss. In contrast, the β-glucan content after secondary soaking for 4 h decreased to 2.40%, representing a more severe loss, which is likely related to the greater exposure of β-glucan to aqueous environments under soaking conditions, promoting its leaching. Overall, the loss of β-glucan was significantly influenced by its water-soluble characteristics and the rate of water migration. The secondary infiltration process can effectively reduce the loss of water-soluble components and better preserve β-glucan content compared with secondary soaking, thereby maintaining the dietary fiber functionality of hulless barley.

Consistent with the above findings, Tufail et al. [41] investigated triple-frequency sono-germination and soaking treatments on barley and reported that soaking alone induced severe leaching of phenolic and flavonoid compounds from barley kernels, which was consistent with our observation that conventional secondary soaking caused greater losses of polyphenols, flavonoids and β-glucan than staged secondary infiltration. Their triple-frequency sono-germination strategy could upregulate the biosynthesis of bioactive compounds and elevate antioxidant activity, but the combined sono-germination treatment relied on sufficient free water uptake to trigger seed germination. Excess free water not only facilitated nutrient leaching but also activated endogenous oxidative and hydrolytic enzymes, resulting in partial degradation of β-glucan and phenolics. Similarly, Tufail et al. [42] also demonstrated that germination combined with ultrasound treatment increased polyphenol and flavonoid contents of highland barley fractions via biological activation. Nevertheless, such germination-based modification inevitably alters the intact kernel morphology and generates sprout tissue, which is not suitable for manufacturing whole pre-gelatinized hulless barley rice products. Compared with the sono-germination approaches in those two studies, our secondary infiltration treatment achieves controlled water permeation without initiating grain germination. By restricting excessive free water penetration into kernels, this method minimizes the activation of polyphenol oxidase and hydrolytic enzymes and mitigates the leaching of water-soluble polyphenols, flavonoids and β-glucan. Therefore, the staged infiltration strategy provides a physical non-germination route to balance starch pre-gelatinization and bioactive component retention for whole hulless barley grains.

### 3.4. Preliminary Screening of the Drying Process

#### 3.4.1. Effects of Different Drying Temperatures and Durations on the Moisture Content of Pre-Gelatinized Hulless Barley Rice

Table 7 shows that different drying temperatures and durations had significant effects on the moisture content of pre-gelatinized hulless barley rice. At 80 °C, the moisture content decreased from 9.67% at 90 min to 6.14% at 150 min, indicating that low-temperature drying over an extended period allowed for slow moisture evaporation. At 100 °C, the moisture content decreased to 5.75% at 90 min and further to 5.34% at 120 min, suggesting that medium-temperature drying was more efficient. At 120 °C, the moisture content was 5.31% at 50 min and decreased to 3.68% at 110 min, approaching the safe moisture content standard. Under high-temperature conditions of 140 °C and 160 °C, moisture evaporation was accelerated; notably, at 160 °C for 70 min, the lowest moisture content of 1.41% was recorded. Through reasonable regulation of the drying process, the moisture content of pre-gelatinized hulless barley rice can be effectively controlled within the safe range, meeting storage and processing requirements.

#### 3.4.2. Effects of Different Drying Temperatures and Durations on the Sensory Scores of Pre-Gelatinized Hulless Barley Rice

**Figure 5:**
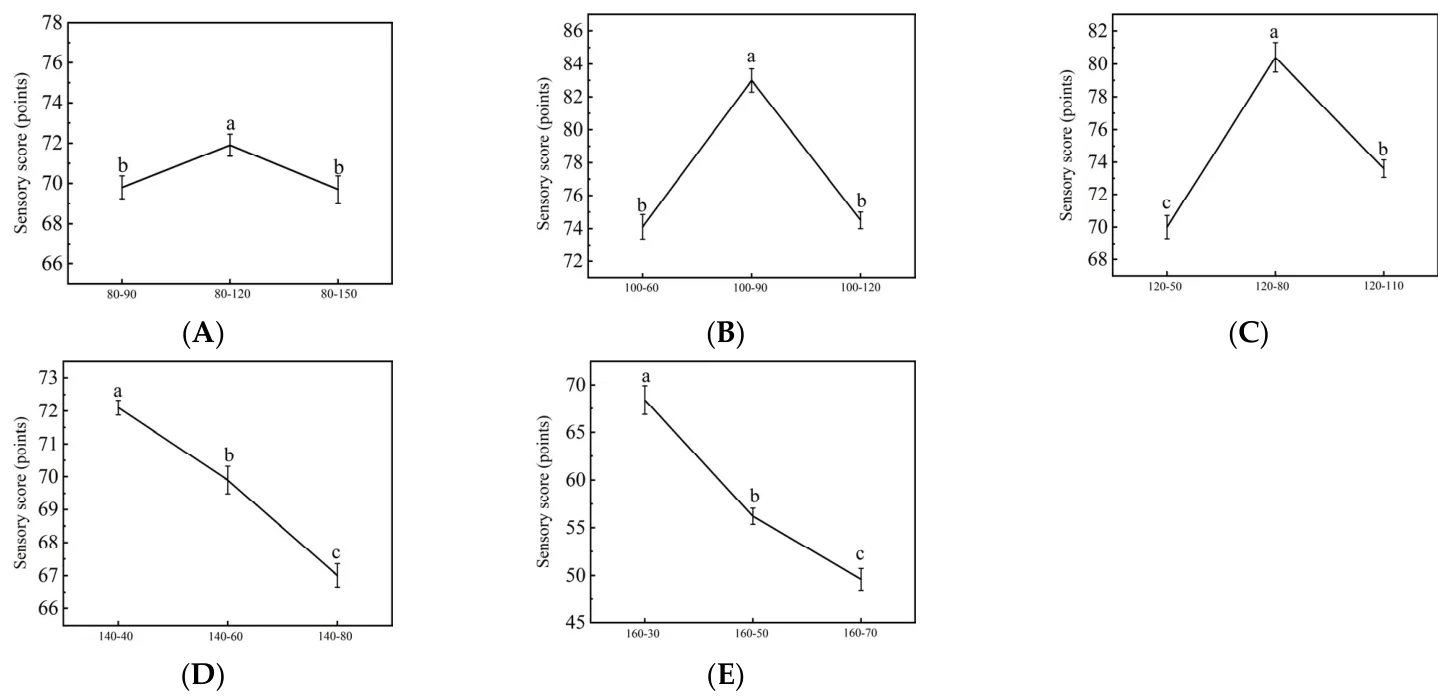
Sensory scores of pre-gelatinized hulless barley rice under different drying processes following secondary infiltration for 4 h. (**A**) Drying at 80 °C, (**B**) drying at 100 °C, (**C**) drying at 120 °C, (**D**) drying at 140 °C, (**E**) drying at 160 °C. Different superscript letters indicate significant differences among groups (*p* < 0.05).

Figure 5 illustrates the changes in the sensory scores of pre-gelatinized hulless barley rice as a function of drying time at different drying temperatures. At 80 °C, the sensory scores initially increased and then decreased, reaching the highest value at 120 min, indicating that appropriate low-temperature drying helps improve mouthfeel, whereas excessively long drying times adversely affect texture. At 100 °C, the highest sensory score was observed at 90 min, suggesting that moderate temperature and time are favorable for maintaining texture and enhancing flavor. At 120 °C, the maximum score occurred at 80 min, with prolonged drying negatively impacting mouthfeel. At 140 °C and 160 °C, the sensory scores gradually decreased with increasing drying time; notably, excessive drying under high-temperature conditions led to significant deterioration of texture and mouthfeel, severely compromising product quality.

#### 3.4.3. Effects of Different Drying Temperatures and Durations on the Gelatinization Degree of Pre-Gelatinized Hulless Barley Rice

**Figure 6:**
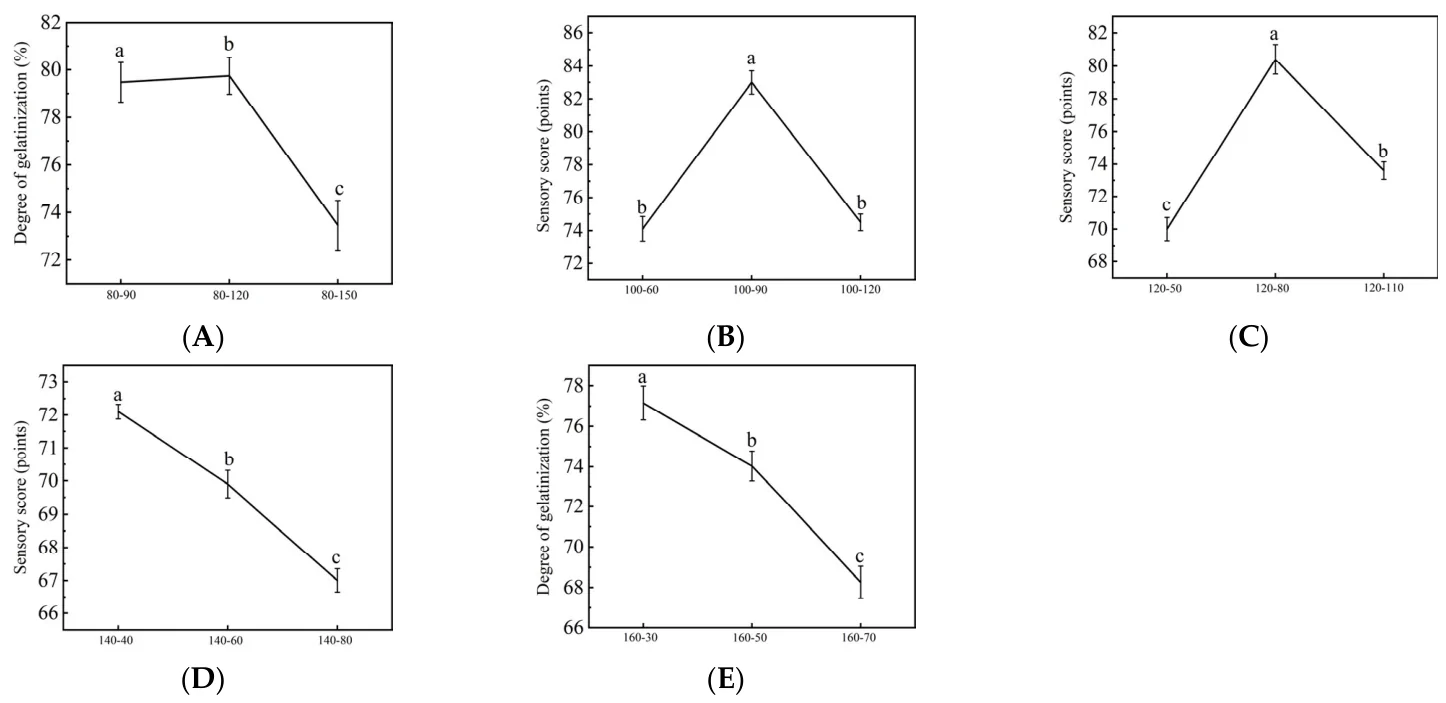
Gelatinization degree of pre-gelatinized hulless barley rice under different drying processes after secondary infiltration for 4 h. (**A**) Drying at 80 °C, (**B**) drying at 100 °C, (**C**) drying at 120 °C, (**D**) drying at 140 °C, (**E**) drying at 160 °C. Different superscript letters indicate significant differences among groups (*p* < 0.05).

Figure 6 illustrates that different drying temperatures and durations significantly affected the gelatinization degree of pre-gelatinized hulless barley rice. Appropriate drying at low and moderate temperatures maintained a relatively high gelatinization degree, whereas prolonged high-temperature treatment tended to cause a decline. At 80 °C, the gelatinization degree reached its maximum at 120 min and decreased after 150 min, indicating that low-temperature drying can preserve gelatinization, but excessive time leads to excessive moisture loss that may affect the starch structure [43]. At 100 °C, the gelatinization degree peaked at 90 min, reaching 87.77%; insufficient drying at 60 min and over-drying at 120 min suggested that 90 min was the optimal drying time. At 120 °C, the gelatinization degree reached 88.98% at 80 min and declined after 110 min, indicating that high-temperature drying requires precise time control to prevent starch structure damage. At 140 °C and 160 °C, the gelatinization degree continuously decreased with prolonged drying time, particularly because rapid moisture evaporation at high temperatures compromised the gelatinization effect.

#### 3.4.4. Effects of Different Drying Temperatures and Durations on the Textural Properties of Pre-Gelatinized Hulless Barley Rice

Table 8 shows that different drying temperatures and durations significantly affected the hardness, springiness, and chewiness of pre-gelatinized hulless barley rice. In general, hardness increased with rising temperature and prolonged time, although low-temperature drying over an extended period could lead to a looser grain structure. At 80 °C, hardness first decreased and then increased, reaching its minimum at 120 min and rebounding after 150 min, indicating that moisture loss influences grain compactness. At 120 °C, hardness increased with time, being relatively low at 50 min and reaching the highest value at 110 min, suggesting that moderate extension of drying time contributes to improved compactness. At 160 °C for 70 min, hardness reached a relatively high level, indicating that high-temperature drying over a prolonged duration exacerbates grain hardening.

The springiness of hulless barley rice showed relatively small variation, with the highest value observed only at 120 °C for 50 min, which may be beneficial for improving mouthfeel. The trend in chewiness was similar to that of hardness, reaching its lowest value at 80 °C for 120 min, indicating that the grains were easier to chew. At 100 °C for 90 min, the chewiness was more favorable, with moderate hardness and a better texture. Above 120 °C, chewiness gradually increased with extended drying time, reaching a relatively high level at 140 °C for 80 min and further increasing at 160 °C for 70 min, suggesting that high-temperature drying over long durations hardens the grain texture, thereby negatively affecting palatability.

#### 3.4.5. Optimal Drying Time at Different Temperatures

Based on a comprehensive analysis of moisture content, sensory scores, gelatinization degree, and textural properties of pre-gelatinized hulless barley rice under different drying temperatures and durations, the optimal drying time at each temperature was determined. At 80 °C for 120 min, the sensory score was the highest, the gelatinization degree remained stable, and the texture was satisfactory, avoiding over-drying caused by prolonged low-temperature exposure. At 100 °C for 90 min, the sensory score peaked, the gelatinization degree reached its maximum, and both hardness and chewiness were optimal, resulting in the best overall mouthfeel. At 120 °C for 80 min, the gelatinization degree approached its highest value, with high drying efficiency and moderate texture. At 140 °C for 40 min, hardening caused by prolonged high-temperature treatment was avoided, and the cooking effect was favorable. At 160 °C for 30 min, the sensory score was optimal, the gelatinization degree steadily increased, and the drying efficiency was the highest, making it suitable for scenarios requiring lower moisture content for storage. Ultimately, the optimal drying temperature and time combinations were determined as follows: 80 °C for 120 min, 100 °C for 90 min, 120 °C for 80 min, 140 °C for 40 min, and 160 °C for 30 min.

### 3.5. Selection of the Optimal Drying Process

Based on the preliminary screening of temperature and time, the optimal drying time corresponding to each temperature was determined as follows: 80 °C for 120 min, 100 °C for 90 min, 120 °C for 80 min, 140 °C for 40 min, and 160 °C for 30 min. On this basis, these five drying parameter combinations were further analyzed and compared.

#### 3.5.1. Sensory Evaluation

**Figure 7:**
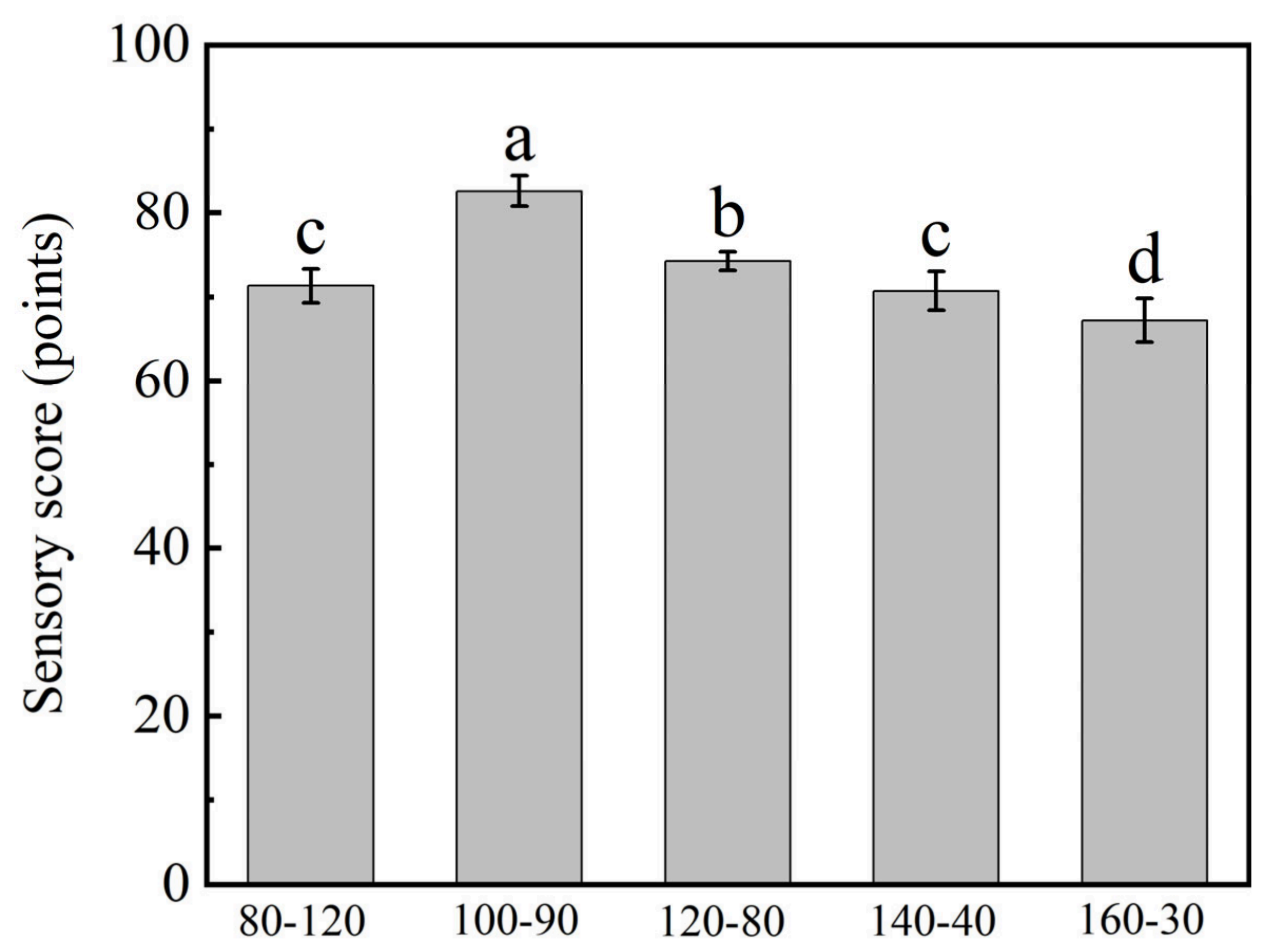
Sensory scores of pre-gelatinized hulless barley rice subjected to different drying temperatures and durations. Different superscript letters indicate significant differences among groups (*p* < 0.05).

Figure 7 illustrates the effects of different drying temperatures and durations on the sensory scores of pre-gelatinized hulless barley rice. Under the treatment of 100 °C for 90 min, the sensory score was the highest, which was significantly superior to the other processes, indicating that the overall texture and mouthfeel of the pre-gelatinized rice were optimal under this condition. This may be attributed to a moderate moisture evaporation rate, which resulted in a uniform internal structure of the grains and a soft yet appropriate mouthfeel [43]. The next best was 120 °C for 80 min, with a slightly lower sensory score but still significantly better than the other conditions, suggesting that appropriately raising the temperature and shortening the drying time can effectively maintain a good mouthfeel and texture. The samples treated at 80 °C for 120 min and 140 °C for 40 min had similar sensory scores, indicating that either low-temperature prolonged drying or relatively high-temperature short-time drying had a greater impact on mouthfeel, possibly causing the grain texture to become hard or loose, thereby affecting eating quality. The sample treated at 160 °C for 30 min had the lowest sensory score, significantly lower than the other treatments, indicating that high-temperature short-time drying may cause excessive drying of the grains, resulting in an overly hard texture or uneven drying, leading to a decline in mouthfeel.

#### 3.5.2. Contents of Flavonoids, Polyphenols, Reducing Sugars, and β-Glucan

**Figure 8:**
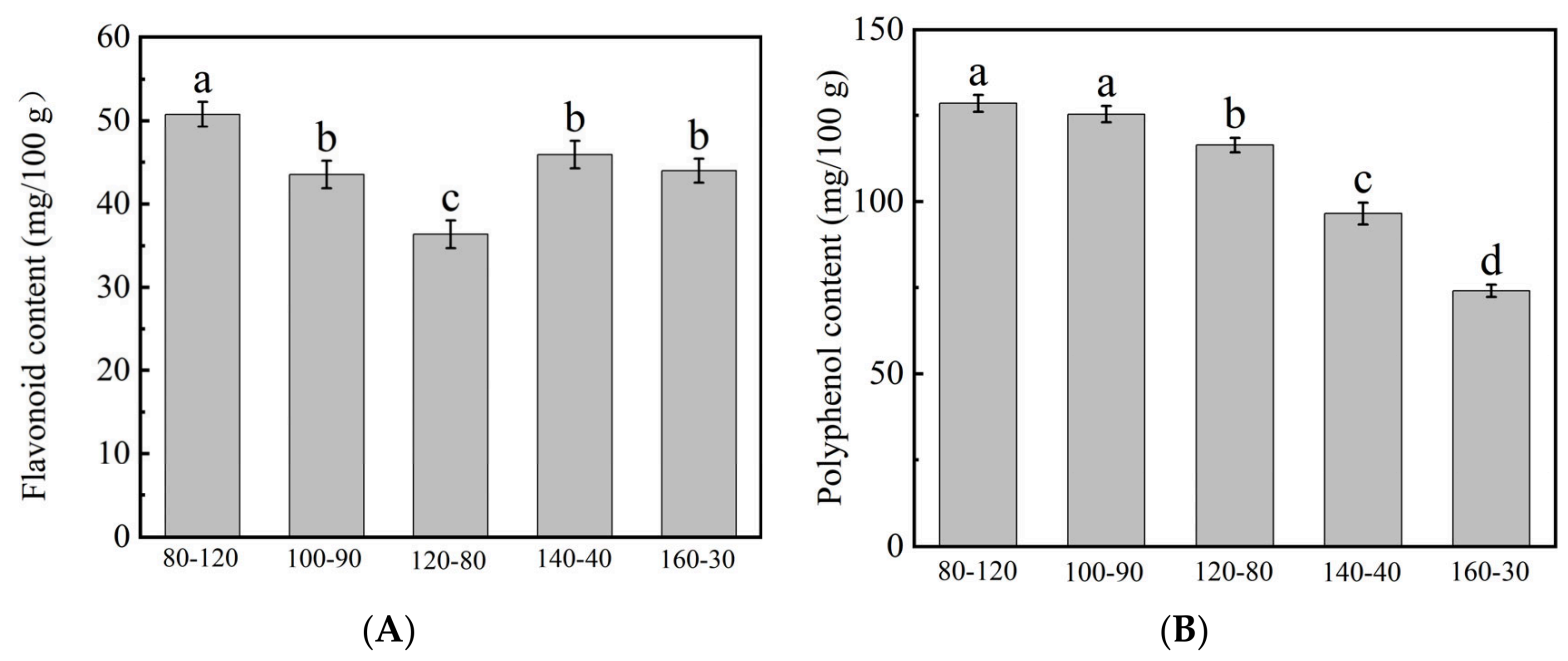

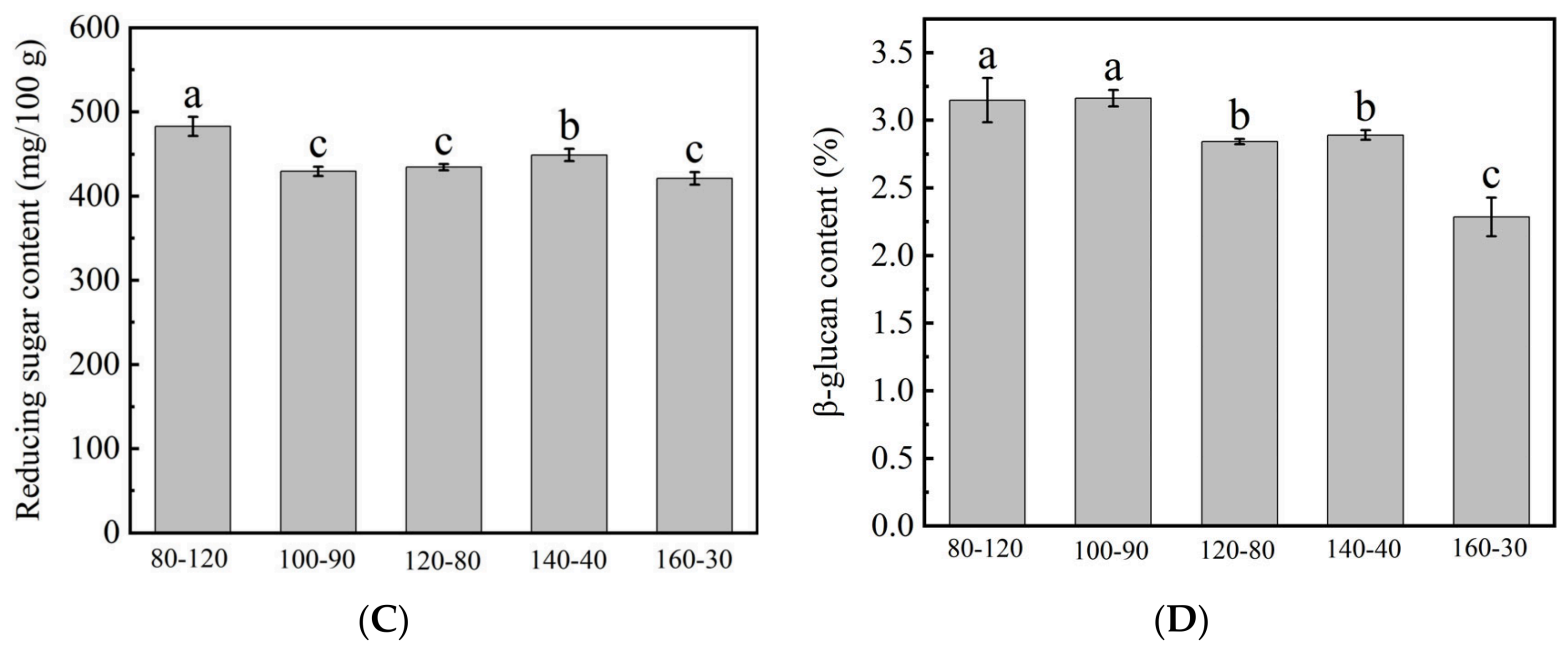
Flavonoid (**A**), polyphenol (**B**), reducing sugar (**C**) and β-glucan (**D**) contents of pre-gelatinized hulless barley rice subjected to different drying temperatures and durations. Different superscript letters indicate significant differences among groups (*p* < 0.05).

Figure 8A illustrates the effects of different drying temperatures and durations on the flavonoid content of pre-gelatinized hulless barley rice. Under the treatment of 80 °C for 120 min, the flavonoid content was the highest, reaching 50.78 mg/100 g, which was significantly higher than that of the other processes, which suggests that low-temperature prolonged drying is beneficial for flavonoid retention. The flavonoid content under 100 °C for 90 min was the second highest, slightly lower than that at 80 °C for 120 min but still remaining at a relatively high level, which suggests that moderately increasing the temperature while controlling drying time can balance flavonoid preservation and processing efficiency under laboratory-scale conditions. At 120 °C for 80 min, the flavonoid content decreased markedly to 36.65 mg/100 g, significantly lower than those at 100 °C for 90 min and 80 °C for 120 min, which implies that further temperature elevation could exacerbate flavonoid degradation. However, under the conditions of 140 °C for 40 min and 160 °C for 30 min, the flavonoid content rebounded to approximately 45 mg/100 g, which suggests that high-temperature short-time drying could reduce the prolonged thermal degradation of flavonoids and improve their retention rate. Overall, the treatment at 80 °C for 120 min maximized flavonoid retention, while 100 °C for 90 min also effectively preserved flavonoid levels with reasonable drying efficiency under laboratory conditions. High-temperature short-time treatment could also effectively reduce flavonoid loss, showing potential for laboratory-scale rapid drying trials.

Figure 8B shows that different drying temperatures and durations significantly affected the polyphenol content of pre-gelatinized hulless barley rice. The overall trend indicated that polyphenol content gradually decreased with increasing drying temperature and shorter drying time. Samples treated at 80 °C for 120 min and 100 °C for 90 min exhibited the highest polyphenol content, reaching 128.46 mg/100 g, which suggests that low or moderate temperature conditions could be more favorable for polyphenol retention. The polyphenol content at 120 °C for 80 min was slightly lower than those of the first two but still remained at a relatively high level of approximately 125.32 mg/100 g, which indicates that moderate temperatures within a certain time range might not significantly destroy polyphenols. After treatment at 140 °C for 40 min, the polyphenol content further decreased to 96.47 mg/100 g, which could be attributed to accelerated thermal degradation at elevated temperatures. The 160 °C for 30 min group had the lowest polyphenol content, at only about 74.04 mg/100 g, which suggests that high-temperature short-time treatment leads to severe polyphenol loss, hypothetically associated with enhanced thermal degradation and oxidation. Comprehensive analysis may suggest that low-temperature prolonged or moderate-temperature drying conditions could be more conducive to the stability of polyphenols in hulless barley at a laboratory scale, while high-temperature short-time treatment significantly reduces their content, which may affect the antioxidant activity and functional properties of the product.

Figure 8C demonstrates that different drying temperatures and durations significantly affected the reducing sugar content of hulless barley. The reducing sugar content at 80 °C for 120 min was the highest, reaching 482.84 mg/100 g, which suggests that low-temperature prolonged treatment could better protect reducing sugars from decomposition. The reducing sugar content at 100 °C for 90 min was slightly lower at about 429.61 mg/100 g, which indicates that moderate temperature and time can preserve reducing sugars well while maintaining efficiency under laboratory conditions. The lowest reducing sugar content was observed at 160 °C for 30 min, at only about 421.14 mg/100 g, which shows that high-temperature, short-time conditions could cause the most significant sugar destruction. In summary, 80 °C for 120 min was the optimal condition for retaining reducing sugars in this laboratory test, while 100 °C for 90 min served as a secondary choice, balancing sugar retention and processing efficiency for lab-scale preparation.

Figure 8D presents the effects of different drying conditions on the β-glucan content of pre-gelatinized hulless barley rice. The results indicated that β-glucan content decreased with increasing temperature and shorter time. Samples treated at 80 °C for 120 min and 100 °C for 90 min had the highest β-glucan content, both exceeding 3.1%, which suggests that low-temperature prolonged treatment could reduce thermal degradation and maintain higher stability. The β-glucan contents at 120 °C for 80 min and 140 °C for 40 min decreased to 2.84% and 2.89%, respectively, which suggests that higher temperatures could accelerate glycosidic bond cleavage and enzymatic degradation. The β-glucan content at 160 °C for 30 min dropped to the lowest level, at only 2.29%, which suggests that high-temperature short-time drying could cause irreversible degradation, and rapid moisture evaporation may further exacerbate its loss. Overall, β-glucan exhibited higher stability under low-temperature, prolonged conditions, with 80 °C for 120 min or 100 °C for 90 min being the optimal drying conditions identified in this laboratory-scale experiment to effectively reduce degradation and preserve the nutritional value of pre-gelatinized hulless barley rice. Further pilot-scale validation will be required to verify the performance of these conditions in larger systems.

#### 3.5.3. DPPH and ABTS^+^ Radical Scavenging Activities

**Figure 9:**
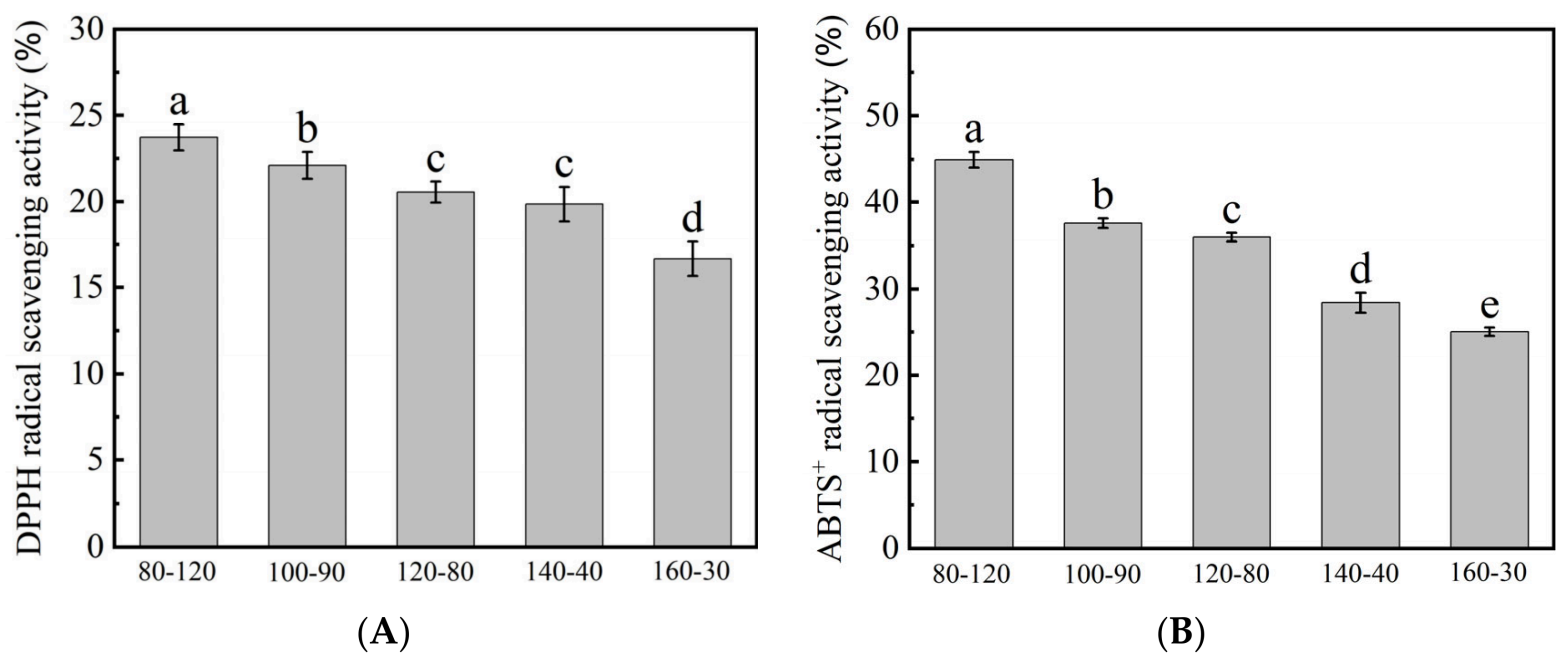
DPPH (**A**) and ABTS^+^ (**B**) radical scavenging activities of pre-gelatinized hulless barley rice subjected to different drying temperatures and durations. Different superscript letters indicate significant differences among groups (*p* < 0.05).

Figure 9A shows that different drying temperatures and durations significantly affected the DPPH radical scavenging activity of hulless barley. The highest scavenging activity was observed at 80 °C for 120 min, reaching 23.74%, possibly because low-temperature, prolonged treatment better preserved the antioxidant components. At 100 °C for 90 min, the activity was slightly lower, at approximately 22.09%, indicating that moderate temperature and time can balance processing efficiency with retention of antioxidant capacity. At 120 °C for 80 min and 140 °C for 40 min, the scavenging activity further decreased to about 19%, likely due to partial degradation of antioxidant components at elevated temperatures. The lowest activity was found at 160 °C for 30 min, at only 16.67%, suggesting that high-temperature short-time treatment caused the most significant damage to antioxidant components [44]. In summary, 80 °C for 120 min was the most effective condition in preserving antioxidant capacity, while 100 °C for 90 min served as a secondary choice, balancing processing efficiency and antioxidant performance.

Figure 9B shows that different drying temperatures and durations significantly affected the ABTS^+^ radical scavenging activity of hulless barley. The highest scavenging activity was observed at 80 °C for 120 min, reaching 44.94%, possibly due to better retention of antioxidant active substances under low-temperature prolonged treatment. The activity at 100 °C for 90 min was slightly lower, at 37.57%, indicating that moderate temperature and time still helped preserve antioxidant capacity. As the temperature further increased, the activity at 140 °C for 40 min decreased to 28.40%, possibly due to partial degradation of antioxidant components at high temperatures. The lowest activity was found at 160 °C for 30 min, at only 25.02%, showing that high-temperature short-time treatment caused the most severe damage to antioxidant components [45]. In summary, 80 °C for 120 min is the optimal condition for maintaining the antioxidant properties of hulless barley, while 100 °C for 90 min is a more ideal choice, as it can improve processing efficiency while retaining antioxidant capacity.

#### 3.5.4. Electronic Tongue Analysis

**Figure 10:**
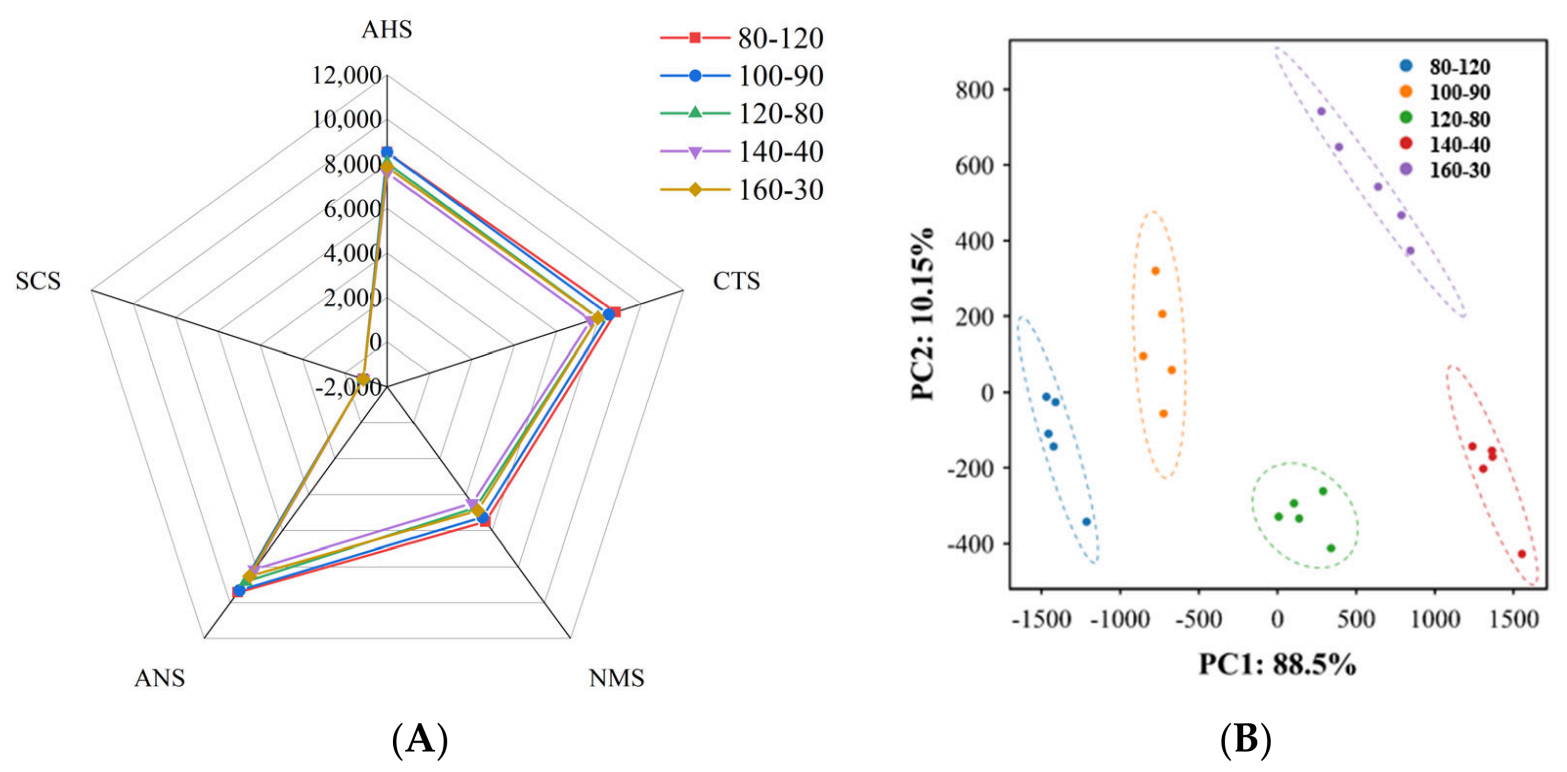
Radar charts (**A**) and PCA plots (**B**) from electronic tongue analysis of pre-gelatinized hulless barley rice subjected to different drying temperatures and durations.

Figure 10A shows that different drying conditions significantly affected the flavor profile of pre-gelatinized hulless barley rice. The responses for sourness, saltiness, umami, and sweetness were prominent, while bitterness was relatively low. The highest sourness was observed with the 160 °C for 30 min treatment, possibly due to the accumulation of acidic compounds, whereas the lowest sourness was found at 80 °C for 120 min, indicating that low-temperature drying reduced the formation of sour substances. The strongest umami was detected at 100 °C for 90 min, suggesting that amino acids and nucleotides were well preserved. Sweetness was higher at 80 °C for 120 min and 100 °C for 90 min, indicating that low or moderate temperatures helped retain free sugars, while the strongest bitterness was observed with the 160 °C for 30 min treatment, possibly due to phenolic oxidation or protein degradation. Principal component analysis (PCA) (Figure 10B) confirmed the flavor differences. The treatments at 80 °C for 120 min and 100 °C for 90 min exhibited similar flavor profiles, with better sweetness and umami and lower sourness and bitterness. The treatments at 120 °C for 80 min and 140 °C for 40 min showed a decline in flavor balance, with slight increases in sourness and bitterness. The flavor profile of the 160 °C for 30 min treatment deviated most significantly, characterized by enhanced sourness and bitterness and reduced sweetness and umami. Comprehensive analysis indicated that 80 °C for 120 min and 100 °C for 90 min best preserved sweetness and umami, resulting in the optimal flavor, while the 160 °C for 30 min treatment caused the most severe flavor deterioration.

#### 3.5.5. Electronic Nose Analysis

**Figure 11:**
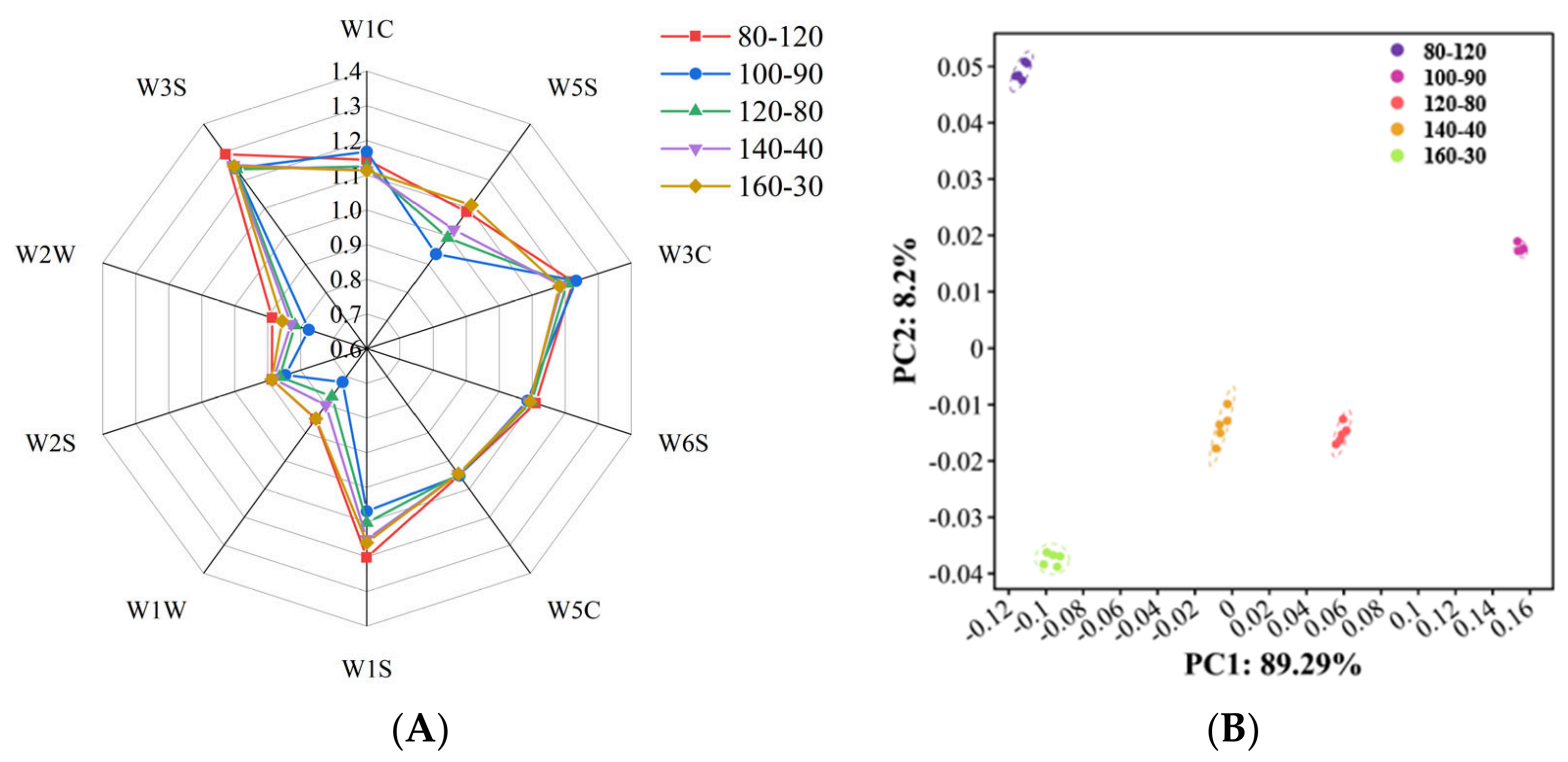
Radar charts (**A**) and PCA plots (**B**) from electronic nose analysis of pre-gelatinized hulless barley rice subjected to different drying temperatures and durations.

Figure 11A shows that different drying conditions significantly affected the volatile compounds of hulless barley. The 80 °C for 120 min treatment exhibited the highest responses in the W1C, W3S, and W5S sensor channels, indicating that this condition favored the retention of aromatic compounds and short-chain volatile organic compounds, thereby preserving the original flavor characteristics of hulless barley. The 100 °C for 90 min treatment showed relatively balanced responses in the W3C, W5C, and W2W channels, suggesting that moderate temperature and time contributed to the stability of volatile substances and avoided excessive degradation. The 120 °C for 80 min and 140 °C for 40 min treatments exhibited higher responses in the W6S and W1S channels, indicating that elevated temperatures promoted lipid oxidation and ester decomposition, while the retention capacity for aromatic compounds decreased. The 160 °C for 30 min treatment showed the lowest responses in most sensor channels, particularly W1C, W3S, and W5S, indicating that high-temperature short-time drying led to extensive degradation of aromatic compounds and short-chain volatile organic compounds. Figure 11B further confirms the differences in the distribution of volatile compounds. The data points of the 80 °C for 120 min treatment were distant from those of the other treatments, indicating the best retention of aroma components. The data points of the 100 °C for 90 min treatment were relatively balanced, indicating that the volatile compounds remained relatively stable. The samples from the 120 °C for 80 min and 140 °C for 40 min treatments clustered together, possibly because oxidation reactions led to similar flavor evolution pathways. The data points of the 160 °C for 30 min treatment deviated the most, indicating that high-temperature short-time drying caused substantial degradation of volatile compounds and pronounced changes in aroma characteristics.

In summary, the 80 °C for 120 min treatment was able to maximize the retention of the original aroma of hulless barley, the 100 °C for 90 min treatment helped maintain the stability of volatile compounds, the 120 °C for 80 min and 140 °C for 40 min treatments exhibited oxidative characteristics, and the 160 °C for 30 min treatment resulted in a substantial loss of aroma components. Therefore, an appropriate combination of temperature and time is the key to optimizing the flavor quality of hulless barley during drying.

In addition, this study has several limitations. First, only one hulless barley cultivar was used in the present experiment; thus, the conclusions obtained may not be directly extrapolated to other cultivars with distinct grain morphological and chemical characteristics. Second, all processing and analytical experiments were carried out at laboratory scale. Pilot-scale production trials are required to evaluate the stability of this infiltration-drying technology before industrial translation. Third, the antioxidant properties were evaluated by in vitro chemical assays. Further in vivo investigations are necessary to verify the physiological bioactivities of pre-gelatinized hulless barley rice. Future work will screen multiple barley varieties, conduct scale-up tests, and explore in vivo biological effects to support the practical application of the pre-gelatinized whole-grain product.

## 4. Conclusions

In this study, the processing technology of pre-gelatinized hulless barley rice was investigated, with a focus on optimizing two critical steps: infiltration and drying. For the infiltration process, the effects of different infiltration cycles and durations were evaluated using sensory scores, textural properties, and degree of gelatinization as the primary indicators. The results demonstrated that the secondary infiltration for 4 h treatment yielded the best performance in terms of mouthfeel, texture, and gelatinization degree. Compared with the traditional soaking process, this infiltration approach significantly reduced the loss of water-soluble nutrients, achieving higher retention rates for polyphenols, flavonoids, and β-glucan, effectively enhancing the nutritional value of the final product.

After determining the optimal infiltration conditions, the corresponding optimal drying times were identified. It was found that the treatments at 80 °C for 120 min and 100 °C for 90 min exhibited the best sensory quality and textural properties. Low-temperature drying was more favorable for retaining polyphenols and flavonoids, and the β-glucan content under these conditions reached about 3.1%. Antioxidant assays further confirmed that the treatments at 80 °C for 120 min and 100 °C for 90 min exhibited the highest DPPH and ABTS^+^ radical scavenging activities. Based on a comprehensive evaluation of sensory, textural, gelatinization, and nutritional functional indicators, the optimal drying conditions for pre-gelatinized hulless barley rice were determined to be 80–100 °C for 90–120 min. This optimized process not only ensures processing efficiency but also maximizes the retention of nutritional and functional properties of hulless barley, which shows promising potential for future large-scale manufacture.

## Figures and Tables

**Table 1:** Sensory scoring criteria for pre-gelatinized hulless barley rice.

Category (Score)	Scoring Criteria	Points
Color (10 points)	Uniform and normal color	8–10
	Relatively uniform, slightly dull	4–7
	Excessively dark or off-color	0–3
Aroma (20 points)	Pronounced natural rice aroma	14–20
	Natural rice aroma, no off-odor	7–13
	Weak aroma or unpleasant odor	0–6
Taste (20 points)	Rich taste	14–20
	Moderate taste	7–13
	Poor taste	0–6
Chewiness (30 points)	Firm yet soft and palatable	21–30
	Moderate firmness and chewiness	11–20
	Hard core or distinctly mushy	0–10
Viscoelasticity (20 points)	Strong viscoelasticity, soft mouthfeel	14–20
	Slightly sticky, moderate elasticity	7–13
	Non-sticky, mushy or undercooked	0–6

**Table 2:** Performance characteristics of electronic tongue sensors.

No.	Sensor Code	Sensor Response Characteristic
1	AHS	Sourness
3	CTS	Saltiness
4	NMS	Umami
6	ANS	Sweetness
7	SCS	Bitterness

**Table 3:** Performance characteristics of electronic nose sensors.

No.	Sensor Code	Sensor Response Characteristic
1	W1C	Sensitive to aromatic compounds
2	W5S	Sensitive to nitrogen oxides
3	W3C	Sensitive to ammonia and aromatic compounds
4	W6S	Selective to hydrides
5	W5C	Sensitive to alkanes and aromatic compounds
6	W1S	Sensitive to methyl compounds
7	W1W	Sensitive to inorganic sulfides
8	W2S	Sensitive to alcohols and some aromatic compounds
9	W2W	Sensitive to aromatic and organic sulfides
10	W3S	Sensitive to long-chain alkanes

**Table 4:** Textural properties of hulless barley under different infiltration cycles and durations.

Process	Hardness/g	Springiness/mm	Chewiness/g
1–1	15,483.91 ± 367.91 ^a^	0.07 ± 0.01 ^b^	136.57 ± 15.40 ^a^
1–2	15,372.17 ± 447.34 ^ab^	0.08 ± 0.01 ^ab^	127.26 ± 6.91 ^ab^
1–3	15,661.08 ± 164.77 ^a^	0.08 ± 0.01 ^ab^	121.75 ± 18.88 ^ab^
1–4	14,886.55 ± 574.69 ^bc^	0.11 ± 0.06 ^a^	128.76 ± 13.57 ^ab^
2–1	14,543.90 ± 391.50 ^c^	0.09 ± 0.01 ^ab^	111.84 ± 16.02 ^bc^
2–2	13,886.60 ± 163.54 ^d^	0.09 ± 0.01 ^ab^	98.27 ± 14.68 ^c^
2–3	10,717.10 ± 593.99 ^e^	0.09 ± 0.01 ^ab^	78.85 ± 15.47 ^d^
2–4	9845.98 ± 120.97 ^f^	0.08 ± 0.01 ^b^	56.53 ± 7.20 ^e^

Note: Within each column, different superscript letters indicate significant differences (*p* < 0.05).

**Table 5:** Basic nutritional component contents of pre-gelatinized hulless barley rice subjected to different processing treatments.

Parameter	Raw Material	Secondary Infiltration	Secondary Soaking
Moisture content (%)	13.49 ± 0.11 ^a^	8.65 ± 0.14 ^b^	8.68 ± 0.08 ^b^
Fat content (%)	1.80 ± 0.04 ^a^	1.78 ± 0.02 ^a^	1.58 ± 0.11 ^b^
Protein content (%)	11.50 ± 0.36 ^a^	10.67 ± 0.15 ^b^	10.23 ± 0.25 ^b^
Starch content (%)	64.80 ± 0.40 ^a^	60.2 ± 0.15 ^b^	60.5 ± 0.10 ^b^

Note: Within each row, different superscript letters indicate significant differences (*p* < 0.05).

**Table 6:** Textural properties of pre-gelatinized hulless barley rice subjected to different processing treatments.

Treatment	Hardness/g	Springiness/mm	Chewiness/g
Raw material	18,290.24 ± 420.52 ^a^	0.10 ± 0.01 ^a^	155.36 ± 19.45 ^a^
Secondary infiltration (4 h)	9845.98 ± 120.97 ^c^	0.08 ± 0.00 ^b^	56.53 ± 7.20 ^b^
Secondary soaking (4 h)	11,937.74 ± 305.24 ^b^	0.10 ± 0.02 ^ab^	137.68 ± 14.42 ^a^

Note: Within each column, different superscript letters indicate significant differences (*p* < 0.05).

**Table 7:** Moisture content of pre-gelatinized hulless barley rice after secondary infiltration for 4 h under different drying conditions.

Drying Condition	Moisture Content (%)	Drying Condition	Moisture Content (%)
80–90	9.67 ± 0.07 ^a^	140–40	4.11 ± 0.04 ^e^
80–120	8.65 ± 0.06 ^b^	140–60	3.67 ± 0.03 ^f^
80–150	6.14 ± 0.07 ^c^	140–80	2.74 ± 0.02 ^g^
100–60	8.49 ± 0.05 ^b^	160–30	6.01 ± 0.04 ^c^
100–90	5.75 ± 0.04 ^cd^	160–50	2.70 ± 0.02 ^g^
100–120	5.34 ± 0.05 ^d^	160–70	1.41 ± 0.01 ^h^
120–50	5.31 ± 0.03 ^d^		
120–80	4.07 ± 0.04 ^e^		
120–110	3.68 ± 0.02 ^f^		

Note: Within each column, different superscript letters indicate significant differences (*p* < 0.05).

**Table 8:** Textural properties of pre-gelatinized hulless barley rice under different drying processes after secondary infiltration for 4 h.

Process	Hardness/g	Springiness/mm	Chewiness/g
80–90	11,568.78 ± 146.31 ^e^	0.1 ± 0.02 ^ab^	113.39 ± 9.92 ^abc^
80–120	9845.98 ± 120.97 ^d^	0.08 ± 0.00 ^c^	56.53 ± 7.2 ^g^
80–150	10,035.85 ± 485.03 ^c^	0.1 ± 0.02 ^d^	112.07 ± 8.5 ^bcd^
100–60	12,489.14 ± 150.44 ^cd^	0.11 ± 0.02 ^ab^	106.6 ± 8.05 ^cd^
100–90	10,709.90 ± 327.69 ^f^	0.09 ± 0.01 ^bc^	74.12 ± 6.44 ^f^
100–120	13,733.01 ± 412.62 ^b^	0.1 ± 0.02 ^ab^	106.82 ± 4.86 ^cd^
120–50	12,510.89 ± 266.52 ^cd^	0.09 ± 0.01 ^abc^	106.73 ± 8.59 ^cd^
120–80	12,342.37 ± 295.88 ^d^	0.1 ± 0.01 ^ab^	113.94 ± 3.32 ^abc^
120–110	15,085.49 ± 316.86 ^a^	0.1 ± 0.01 ^ab^	110.58 ± 5.12 ^bcd^
140–40	11,644.09 ± 402.92 ^e^	0.09 ± 0.01 ^bc^	87.52 ± 13.2 ^e^
140–60	13,673.73 ± 494.58 ^b^	0.1 ± 0.01 ^abc^	120.28 ± 6.92 ^ab^
140–80	13,795.48 ± 443.69 ^b^	0.09 ± 0.01 ^bc^	121.62 ± 12.44 ^ab^
160–30	12,671.29 ± 375.20 ^cd^	0.11 ± 0.02 ^ab^	99.83 ± 8.66 ^d^
160–50	12,775.43 ± 472.75 ^cd^	0.11 ± 0.02 ^ab^	109.37 ± 9.04 b^cd^
160–70	13,700.40 ± 346.95 ^e^	0.12 ± 0.02 ^a^	125.21 ± 10.02 ^a^

Note: Within each column, different superscript letters indicate significant differences (*p* < 0.05).

## Data Availability

The data presented in this study are available on request from the corresponding authors. The raw sensory evaluation data involving human panelists are not publicly available due to privacy restrictions.
